# Phytochemical Study of Stem and Leaf of *Clausena lansium*

**DOI:** 10.3390/molecules24173124

**Published:** 2019-08-28

**Authors:** Wenwen Peng, Xiaoxiang Fu, Yuyan Li, Zhonghua Xiong, Xugen Shi, Fang Zhang, Guanghua Huo, Baotong Li

**Affiliations:** 1 Jiangxi Key Laboratory for Conservation and Utilization of Fungal Resources, Jiangxi Agricultural University, Nanchang 330045, China; 2 College of Agriculture, Jiangxi Agricultural University, Nanchang 330045, China; 3 School of Land Resources and Environment, Jiangxi Agricultural University, Nanchang 330045, China

**Keywords:** *Clausena lansium*, aromatic glycosides, sesquiterpene glycosides, dihydrofuranocoumarin glycosides

## Abstract

*Clausena lansium* Lour. Skeels (Rutaceae) is widely distributed in South China and has historically been used as a traditional medicine in local healthcare systems. Although the characteristic components (carbazole alkaloids and coumarins) of *C. lansium* have been found to possess a wide variety of biological activities, little attention has been paid toward the other components of this plant. In the current study, phytochemical analysis of isolates from a water-soluble stem and leaf extract of *C. lansium* led to the identification of 12 compounds, including five aromatic glycosides, four sesquiterpene glycosides, two dihydrofuranocoumarin glycosides, and one adenosine. All compounds were isolated for the first time from the genus *Clausena*, including a new aromatic glycoside (**1**), a new dihydrofuranocoumarin glycoside (**6**), and two new sesquiterpene glycosides (**8** and **9**). The phytochemical structures of the isolates were elucidated using spectroscopic analyses including NMR and MS. The existence of these compounds demonstrates the taxonomic significance of *C. lansium* in the genus *Clausena* and suggests that some glycosides from this plant probably play a role in the anticancer activity of *C. lansium* to some extent.

## 1. Introduction

**Figure 1:**
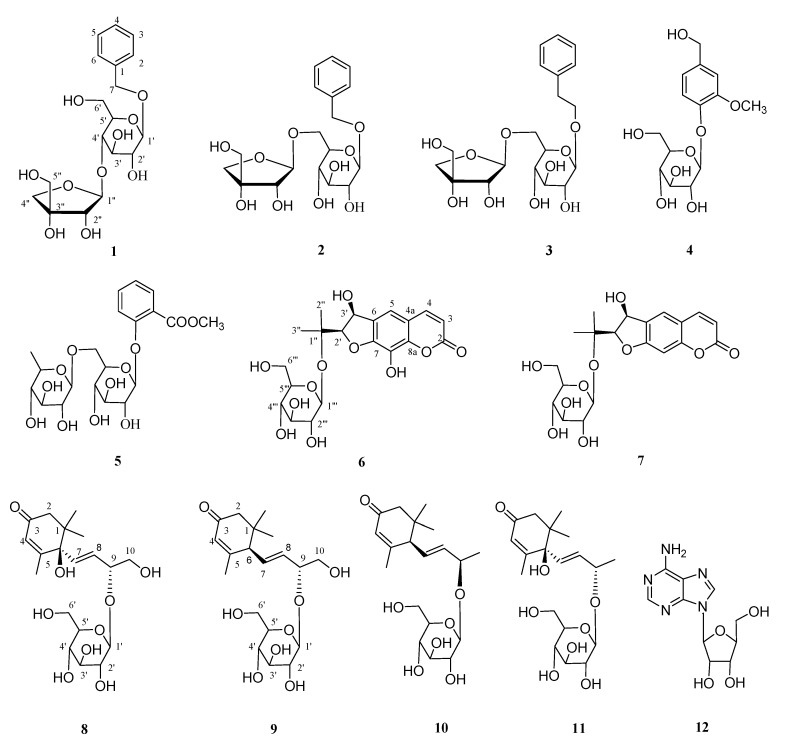
The chemical structures of compounds **1**–**12** from the stem and leaf of *Clausena lansium.*

The genus *Clausena* (Rutaceae) is comprised of approximately 30 species that are scattered throughout the subtropical and tropical regions, including China, Vietnam, Indonesia, Malaysia, and the Philippines [1,2]. There are approximately 10 species as well as 2 varieties in China, which appear in Southern China. *Clausena lansium* Lour. Skeels (Rutaceae), belonging to the genus *Clausena* of the family Rutaceae, is a fruit tree and a species of strongly scented evergreen tree growing in South China [1,2]. *C. lansium* is famous for their fruits, which are usually very popular tropical, health-promoting fruits, while their roots, stems, leaves, and seeds have also been extensively applied in folk medicine or traditional Chinese medicine for the treatments of abdominal pain, malaria, cold, dermatopathy, and snake bites [2,3]. Various biological studies, on the alkaloids, coumarins, and sesquiterpenes from this plant have reported the neuroprotective [4,5], antitumor [6,7], hepatoprotective [8], anti-inflammatory [9], antifungal [10], antioxidant [11], antiobesity [12], nematicidal [13], antimicrobial [14], and hypoglycemic [15] effects of the *C. lansium*. In our previous studies, some carbazole alkaloids [16] and coumarins [17] were separated from the stem and leaf of *C. lansium*. As a part of our ongoing research into the natural products possessing structural and biological diversity from *C. lansium*, a systematic phytochemical study on the stem and leaf of *C. lansium* was accordingly carried out. The investigation resulted in the separation and characterization of five aromatic glycosides (**1**–**5**), two dihydrofuranocoumarin glycosides (**6**,**7**), four sesquiterpene glycosides (**8**–**11**), and one adenosine (**12**) (Figure 1). All compounds were isolated for the first time from the genus *Clausena*, including a new aromatic glycoside (**1**), a new dihydrofuranocoumarin glycoside (**6**), and two new sesquiterpene glycosides (**8** and **9**). The molecular structures of new compounds were established using comprehensive spectroscopic studies. The known compounds were determined by comparing their experimental data with those described in the literature. The existence of these compounds demonstrates the taxonomic significance of *C. lansium* in the genus *Clausena* and suggests that some glycosides from this plant probably play a role in the anticancer activity of *C. lansium* to some extent.

## 2. Results and Discussion

### 2.1. Elucidation of Chemical Structures of Four New Compounds **1**,**6**,**8**,**9**

**Figure 2:**
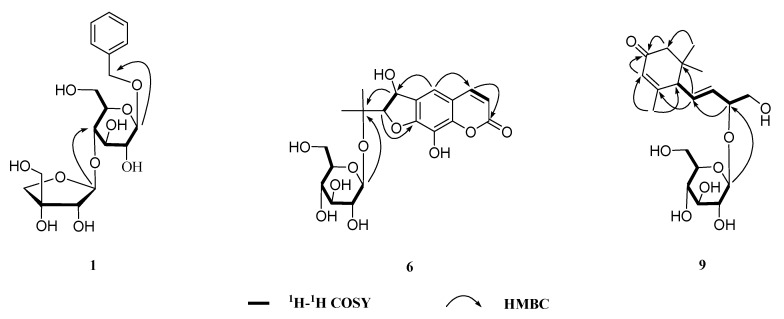
Some key HMBC and ^1^H–^1^H COSY correlations of **1**, **6,** and **9**.

Claulanaroside (**1**) was obtained as a white amorphous powder, and its elemental composition was determined to be C_18_H_26_O_10_ by HRESIMS *m/z*: 425.1623 (Calculated for: [C_18_H_26_O_10_ + Na]^+^, 425.1628) with 6 degrees of unsaturation. The Infrared Radiation (IR) spectrum exhibited absorptions for hydroxyl groups (3441 and 1062 cm^−1^) and the aromatic ring (1618, 1547, and 1498 cm^−1^). The ^1^H NMR data of **1** (Table 1) showed clear signals for five aromatic protons (*δ*_H_ 7.31 (2H, d, *J* = 7.2 Hz), 7.23 (2H, m), and 7.18 (1H, d, *J* = 7.3)), indicating a monosubstituted benzene ring in **1**. The ^13^C-NMR spectroscopic data (Table 1) showed the presence of a six-carbon sugar (*δ*_C_ 100.8, 77.2, 76.6, 76.5, 70.3, and 61.4), a five-carbon sugar (*δ*_C_ 109.3, 79.2, 77.6, 73.9, and 64.6), a benzene ring (*δ*_C_ 137.6, 127.8, 127.8, 127.9, 127.9, and 127.3) and an oxymethylene (*δ*_C_ 70.4). Coupled with the above evidence, comparison of the ^1^H- and ^13^C NMR data of **1** and icariside F2 [18] implied that the aglycone of **1** was methylbenzene and the C-7 position was glycosylated. Acid hydrolysis of **1** afforded d-glucose and d-apiose, which were detected by derivatization and HPLC analysis [19,20]. The anomeric configurations of monosaccharide units were confirmed to be β for the d-glucose and d-apiose according to their ^3^*J*_H1-H2_ coupling constants (7–8 and 2–3 Hz, respectively) [18,21,22]. Detailed comparison of the NMR data of **1** with those of icariside F2 revealed that the positions of the β-d-apiofuranosyl linkage were different in **1** and icariside F2. The β-d-apiofuranosyl was located at C-4′ of the β-d-glucopyranosyl of **1** from the HMBC spectrum (Figure 2), which showed that H-1″ (*δ*_H_ 5.27) was correlated to C-4′ (*δ*_C_ 76.6), according to the β-d-glucopyranosylation-induced downfeld shift on the α-carbon [23]. The above deduction was further supported by downfeld shift observed for C-4′ (*δ*_C_ 76.6 ppm) and upfeld shift for C-6′ (*δ*_C_ 61.4 ppm) in **1**. Consequently, compound **1** was identified as a methylbenzene-7-*O*-β-d-apiofuranosyl-(1→4)-*O*-β-d-glucopyranoside and named as claulanaroside, as shown in Figure 1.

Claulancoumside (**6**) was obtained as a white powder crystallization with a blue fluorescence under the ultraviolet lamp (254 nm). The structure of **6** was deduced for one new dihydrofuranocoumarin glycoside mainly by its blue fluorescence and experimental data. In the ^1^H NMR spectrum (Table 2), the resonance characteristics for a cis-double bond (*δ*_H_ 7.91 (1H, d, *J* = 9.5 Hz) and 6.25 (1H, d, *J* = 9.5 Hz)) and a singlet aromatic proton (*δ*_H_ 7.21 (1H, s)), coupled with the blue fluorescence, suggested the presence of a trisubstituted coumarin skeleton in **6 [24,25,26]**. The ^13^C-NMR spectrum (Table 2) of **6** showed 20 C-atom signals, including a coumarin skeleton (*δ*_C_ 161.3, 150.1, 144.9, 144.1, 129.3, 127.8, 114.3, 113.8, and 110.7), a six-carbon sugar (*δ*_C_ 97.2, 76.1, 75.6, 73.1, 69.3, and 59.9), two oxymethines (*δ*_C_ 91.5 and 71.1), two methyls (*δ*_C_ 23.3 and 22.2), and an oxygenated quaternary carbon (*δ*_C_ 77.5 (s)). The ^1^H and ^13^C NMR data of **6** and 8-hydroxysmyrindiol [24] demonstrated that the aglycone of **6** was equivalent to 8-hydroxysmyrindiol and the C-1″ position of **6** was glycosylated, which was further confirmed by the HMBC correlation (Figure 2) from H-1‴ to C-1″ and the downfeld shift observed for C-1″ (*δ*_C_ 77.5 ppm) in **6 [23]**. Acid hydrolysis and HPLC analysis of **6** afforded a d-glucose [19,20]. The anomeric configuration of the d-glucose was confirmed to be β according to its large ^3^*J*_H1-H2_ coupling constants (*J* = 7.5 Hz) [25,26]. In addition, coupled with the ^1^H-^1^H COSY (Figure 2) correlations of H-2′/H-3′, a large vicinal coupling constant (6.5 Hz) of two doublets at *δ*_H_ 4.57 (H-2′) and 5.35 (H-3′) supported the cis orientation. The absolute configurations of **6** at C-2′ and C-3′ were established by comparing its specific rotation with 1′-*O*-β-d-glucopyranosyl-(2*S*,3*R*)-3-hydroxynodakenetin (${\left[\alpha \right]}_{D}^{20}$−14.0° (pyridine; *c* 0.5)) [25] and 1′-*O*-β-d-glucopyranosyl (2*R*,3*S*)-3-hydroxynodakenetin (${\left[\alpha \right]}_{D}^{21}$ +15.1° (MeOH; *c* 0.05)) [26]. With a specific rotation value of +24.3 (MeOH; *c* 1.1), compound **6** was assigned to the 2′*R*,3′*S* configurations. Therefore, compound **6** was deduced as a 1″-*O-*β-d-glucopyranosyl (2′*R*,3′*S*)-3′,8-dihydroxyarmesin and named as claulancoumside, as shown in Figure 1.

Clausesquiside A (**8**) was obtained as an amorphous powder with ${\left[\alpha \right]}_{D}^{20}$ −113.4° (MeOH; c 0.68). The UV spectrum showed absorption maxima at 237 nm. The IR spectrum indicated the presence of carbonyl (1668 cm^−1^) and hydroxyl (3447 and 1043 cm^−1^) groups. The ^1^H NMR spectrum of **8** (Table 3) showed two trans-olefinic protons (*δ*_H_ 6.92 (1H, dd, *J* = 15.6 Hz) and 5.79 (1H, dd, *J* = 15.6, 7.4 Hz)), one olefinic proton singlet (*δ*_H_ 5.85 (1H, s)), and three methyl singlets (*δ*_H_ 1.91 (3H, s), 0.95 (3H, s), and 0.93 (3H, s)). The ^13^C NMR spectrum of **8** (Table 3) exhibited a ketonic carbonyl at *δ*_C_ 199.5; four olefinic carbons at *δ*_C_ 165.3, 133.9, 127.0, and 125.3; an oxymethine at *δ*_C_ 77.6; an oxygenated quaternary carbon at *δ*_C_ 78.4; an oxymethylene at *δ*_C_ 64.1; three methyls at *δ*_C_ 22.9, 21.6, and 17.7; a quaternary carbon at *δ*_C_ 40.5; a methylene at *δ*_C_ 48.8; and a six-carbon sugar at *δ*_C_ 99.5, 76.2, 76.2, 73.0, 69.6, and 60.8. The ^1^H and ^13^C NMR spectral data of **8** were very similar to those of (6*R*,9*R*)-roseoside [27], differing only in the presence of an oxymethylene at *δ*_C_ 64.1 in **8**, instead of a methyl at *δ*_C_ 21.2 that is found in (6*R*,9*R*)-roseoside. The absolute configuration at the 6-position in **8** was determined to be *R*, judging from the negative and positive Cotton effects at 241 and 322 nm, respectively, in the CD spectrum [27]. The β-d-glucopyranosylation-induced shift-trend rule suggested the absolute configuration at the 9-position of **8** to be *R* [23,28], which was further supported by the remarkable chemical shift difference between C-7 (*δ*_C_ 133.9) and C-8 (*δ*_C_ 127.0) of **8,** as shown in [27]. So, compound **8** was elucidated as a (6*R*,9*R*,4*Z*,7*E*,)-6,9,10-trihydroxy-4,7-megastigmadien-3-one-9-*O*-β-d-glucopyranoside and named as clausesquiside A, as shown in Figure 1.

Clausesquiside B (**9**), a yellowish amorphous powder, possessed the virtually identical NMR (Table 3) and MS data as opuntiside A [29]. Its plane structure is determined by the HMBC and ^1^H-^1^H COSY spectrums (Figure 2). The CD data (321 (Δ*ε* –7.1), 254 (Δ*ε* +186.2) nm) showed a positive maximum at 254 nm, which was identical to the CD data of opuntiside A, indicating that the C-6 of **9** had an absolute *R*-configuration. According to the β-d-glucopyranosylation-induced shift-trend rule [23,28], the absolute configuration of C-9 was deduced for *R* from the downfeld shift observed for C-9 (*δ*_C_ 77.6 ppm). Based upon the results of the combined spectroscopic analyses, the structure of this compound **9** was established as (6*R*,9*R*,4*Z*,7*E*)-9,10-dihydroxy-4,7-megastigmadiene-3-one-9-*O*-β-d-glucopyranoside and named as clausesquiside B. As a result, the absolute structure of **9** at C-9 was substantiated for the first time in this study.

### 2.2. Structural Identification and Function of the Known Compounds **2**–**5**,**7**,**10**–**12**

Comparing the experimental data of the known compounds with those described in the literatures, the phytochemical structures of the eight known compounds were identified as: Icariside F2 (**2**) [18], Icariside D1 (**3**) [30], Vanilloloside (**4**) [31], methyl benzoate-2-(6-*O*-α-l-rhanmopyranosyl)-*O*-β-d-glucopyranoside (**5**) [32], 1′-*O*-β-d-glucopyranosyl (2*S*,3*R*)-3-hydroxyarmesin (**7**) [24], (6*R*,9*R*,4*Z*,7*E*)-9,10-dihydroxy-4,7-megastigmadien-3-one-9-*O*-β-d-glucopyranoside (**10**) [33], (6*R*,9*S*)-Roseoside (**11**) [27], and adenosine (**12**) [34]. The eight known compounds were obtained from the genus *Clausena* for the first time, but the aglycone analogs of compounds **2**–**5**,**10**,**11** have been isolated from *Clausena excavata* [35,36] in our previous research about the phytochemical constituents of the genus *Clausena*, which may exhibit the chemical relationship between *C. lansium* and *C. excavata*. In addition, the analogs of compound **7** and its aglycone have been previously isolated from three different families such as *Angelica archangelica* (Apiaceae) [25], *Pleurospermum rivulorum* (Apiaceae) [26], *Ferulago asparagifolia* (Apiaceae) [37], *Notopterygium incisum* (Apiaceae) [38], *Glehnia littoralis* (Apiaceae) [39], *Streblus indicus* (Moraceae) [40]*, Dorstenia brasiliensis* (Moraceae) [41]*,* and *Aegle marmelos* (Rutaceae) [24], which may revealed a genetic relationship between the Apiaceae, Moraceae, and Rutaceae families. To the best of our knowledge, the phytochemical constituents of the plant are mainly affected by the genetic and environmental factors during plant growth, extraction, and isolation. Therefore, further phytochemical study on the water-soluble extract of *C. lansium* from different regions should be performed to achieve a full understanding of the chemical composition of *C. lansium*. As a predictable result, more and more chemical constituents will be used as evidence to support the taxonomic significance of *C. lansium* in the genus *Clausena*.

As mentioned in the introduction, as the characteristic components of the genus *Clausena*, the carbazole alkaloids and coumarins have been found to possess a variety of structures and biological activities [42,43]; however, little attention has been paid toward the other components of this plant, especially the water-soluble components. The current study demonstrates the presence of the aromatic glycosides, sesquiterpene glycosides, and coumarin glycosides. The carbazole alkaloids are considered to be the main anti-cancer components of the genus *Clausena* [6,7,44,45,46]. However, several reports demonstrated that the analogs of aromatic glycosides [47,48] and sesquiterpene glycosides [49] also possessed a certain degree of anti-cancer activity. Therefore, it is suggested that some glycosides from this plant probably play a role in the anticancer activity of *C. lansium* to some extent. At the same time, this also provides some clues about testing anticancer activity and expanding the biological scope of this work for our future research.

### 2.3. Characterization of Compounds **1**–**12**

*Claulanaroside* (**1**): UV (MeOH) *λ*_max_ (log *ε*): 218 (6.31), 265 (6.00), 294(5.76) nm; IR (KBr) *ν*_max_ 3441, 1618, 1547, 1498, 1248, 1062 cm^−1^; NMR data (SI 1–SI 5 in Supplementary Material) found in Table 1; positive ESIMS *m/z*: 425 [M + Na]^+^; HRESIMS *m/z*: 425.1623 (Calculated for: [C_18_H_26_O_10_ + Na]^+^, 425.1628).

*Icariside F2* (**2**): ESIMS *m/z*: 425 [M + Na]^+^; ^1^H-NMR (400 MHz, MeOD): *δ* 7.44 (2H, d, *J* = 7.2 Hz, H-3,5), 7.35 (2H, m, H-2,6), 7.30 (1H, m, H-4), 5.08 (1H, d, *J* = 2.6 Hz, H-1″), 4.93 (1H, d, *J* = 12.0 Hz, H-7a), 4.66 (1H, d, *J* = 12.0 Hz, H-7b), and 4.35 (1H, d, *J* = 7.4 Hz, H-1′). ^13^C NMR (101 MHz, MeOD) *δ*: 137.5 (s, C-1), 128.0 (d, C-2,6), 127.9 (d, C-3,5), 127.4 (d, C-4), 70.4 (t, C-7), 101.8 (d, C-1′), 73.6 (d, C-2′), 76.7 (d, C-3′), 70.5 (d, C-4′), 75.6 (d, C-5′), 67.3 (d, C-6′), 109.6 (d, C-1″), 76.6 (d, C-2″), 79.2 (s, C-3″), 73.7 (d, C-4″), and 64.2 (d, C-5″) (NMR spectrogram SI 6–SI 7 in Supplementary Material).

*Icariside D1* (**3**): ESIMS *m/z*: 439 [M + Na]^+^; ^1^H-NMR (400 MHz, MeOD): *δ*: 7.29 (2H, *J* = 7.8 Hz, H-2,6), 7.28 (2H, m, H-3,5), 7.18 (1H, m, H-4), 5.04 (1H, d, *J* = 2.5 Hz, H-1″), 4.62 (1H, d, *J* = 7.7 Hz, H-1′), 4.31 (1H, m, H-8a), 3.83 (1H, m, H-8b), and 3.00 (2H, t, *J* = 7.3 Hz, H-7); ^13^C NMR (101 MHz, MeOD) *δ*: 138.2 (s, C-1), 127.5 (d, C-2,6), 128.1 (d, C-3,5), 125.3 (d, C-4), 35.4 (d, C-7), 69.9 (d, C-8), 102.5 (d, C-1′), 73.2 (d, C-2′), 76.2 (d,C-3′), 69.8 (d, C-4′), 75.0 (d, C-5′), 66.8 (d, C-6′), 109.1 (d, C-1″), 76.1 (d, C-2″), 78.6 (s, C-3″), 73.1 (d, C-4″), and 63.7 (d, C-5″) (NMR spectrogram SI 8–SI 9 in Supplementary Material).

*Vanilloloside* (**4**): ESIMS *m/z*: 339 [M + Na]^+^; ^1^H-NMR (400 MHz, MeOD): *δ*: 7.14 (lH, d, *J* = 8.2 Hz, H-5), 7.04 (lH, d, *J* = 2.1 Hz, H-2), 6.89 (lH, dd, *J* = 2.1, 8.2 Hz; H-6), 4.63 (1H, d, *J* = 7.4 Hz, H-1′), 4.56 (2H, s; H-7), and 3.97 (3H, s, OCH_3_); ^13^C NMR (101 MHz, MeOD) *δ*: 136.3 (s, C-1), 111.2 (d, C-2), 149.4 (s, C-3), 145.8 (s, C-4), 116.5 (d, C-5), 119.3 (d, C-6), 63.6 (t, C-7), 101.5 (d, C-1′), 76.8 (d, C-2′), 76.4 (d, C-3′), 73.5 (d, C-4′), 69.9 (d, C-5′), 61.1, (d, C-6′), and 55.3 (Q, OCH_3_) (NMR spectrogram SI 10–SI 14 in Supplementary Material).

*Methyl benzoate 2-(6-O-α-l-rhanmopyranosyl)-O-β-d-glucopyranoside* (**5**): ESIMS *m/z*: 483 [M + Na]^+^; ^1^H NMR (400 MHz, MeOD) *δ*: 7.79 (1H, dd, *J* = 7.8, 1.6 Hz, H-3), 7.59 (1H, ddd, *J* = 8.4, 7.8, 1.6 Hz, H-5), 7.36 (1H, d, *J* = 8.4 Hz, H-6), 7.17 (1H, dd, *J* = 7.8, 7.8 Hz, H-4), 4.90 (1H, d, *J* = 7.3 Hz, H-1′), 4.67 (1H, d, *J* = 1.3 Hz, H-1″), 3.92 (1H, d, *J* = 9.8 Hz, Hb-6′), 3.91 (3H, s, H-8), 3.81 (1H, m, H-2″), 3.77 (1H, dd, *J* = 9.7, 3.4 Hz, H-3″), 3.70 (2H, m, Ha-6′, H-5″), 3.64 (2H, m, H-2′, H-4′), 3.62 (1H, t, *J* = 8.9 Hz, H-3′), 3.50 (1H, t, *J* = 9.1 Hz, H-5′), 3.35 (1H, t, *J* = 9.6 Hz, H-4″), and 1.14 (1H, d, *J* = 6.2 Hz, H-6″); ^13^C NMR (101 MHz, MeOD) *δ*: 157.2 (s, C-1), 120.9 (d, C-2), 130.8 (d, C-3), 122.3 (d, C-4), 133.8 (d, C-5), 117.5 (d, C-6), 167.1 (s, C-7), 102.3 (d, C-1′), 73.5 (d, C-2′), 75.8 (d, C-3′), 71.0 (d, C-4′), 76.2 (d, C-5′), 66.53 (t, C-6′), 100.8 (d, C-1″), 68.5 (d, C-2″), 70.8 (d, C-3″), 72.6 (d, C-4″), 70.0 (d, C-5″), 16.53 (q, C-6″), and 51.43 (q, OCH_3_) (NMR spectrogram SI 15–SI 16 in Supplementary Material).

*Claulancoumside* (**6**): ${\left[\alpha \right]}_{D}^{21}$ +24.3 (MeOH; *c* 1.1), UV (MeOH) *λ*_max_ (log *ε*): 325 (4.18), 256 (3.53), 223(4.18) nm; IR (KBr, *ν*_max_, cm^−1^): 3341, 2903, 2834, 1702, 1618, 1527, 1471; NMR data (SI 17–SI 21 in Supplementary Material) found in Table 2; positive ESIMS *m/z*: 463 [M + Na]^+^.

*1′-O-β-d-Glucopyranosyl (2S,3R)-3-hydroxyarmesin* (**7**): ESIMS *m/z*: 447 [M + Na]^+^; ^1^H NMR (400 MHz, MeOD) *δ*: 7.79 (1H, d, *J* = 9.6 Hz, H-4), 7.45 (1H, s, H-5), 6.70 (1H, s, H-8), 6.03 (1H, d, *J* = 9.6 Hz, H-3), 5.02 (1H, d, *J* = 6.2 Hz, H-3′), 4.31 (1H, d, *J* = 6.2 Hz, H-2′), 4.31 (1H, d, *J* = 7.8 Hz, H-1‴), 3.14 (2H, m, H-6‴), 2.94 (1H, d, *J* = 6.2 Hz, H-3‴), 2.83 (1H, br s, H-5‴), 2.82 (1H, m, H-4‴), 2.66 (1H, m, H-1‴), 1.23 (3H, s, H-3″), and 1.22 (3H, s, H-2″); ^13^C NMR (101 MHz, MeOD) *δ*: 160.9 (s, C-2), 112.2 (d, C-3), 145.4 (d, C-4), 113.3 (s, C-4a), 126.1 (d, C-5), 129.1 (s, C-6), 162.8 (s, C-7), 98.2 (d, C-8), 156.5 (s, C-8a), 92.3 (d, C-2′), 70.6 (d, C-3′), 78.0 (s, C-1″), 25.1 (q, C-2″), 23.3 (q, C-3″), 97.8 (d, C-1‴), 73.9 (d, C-2‴), 77.4 (d, C-3‴), 70.3(d, C-4‴), 77.1 (d, C-5‴), and 61.3 (t, C-6‴) (NMR spectrogram SI 22–SI 26 in Supplementary Material).

*Clausesquiside A* (**8**): ${\left[\alpha \right]}_{D}^{20}$ −113.4° (MeOH; c 0.68); UV (MeOH) *λ*_max_ (log *ε*): 237 (3.21); CD (*c* 0.0042, MeOH) Δ*ε* (*λ* nm): −15.6 (241) and +0.8 (322) nm; IR (KBr, *ν*_max_, cm^−1^): 3447, 2981, 1668, 1105, 1043, 961; NMR data (SI 27–SI 28 in Supplementary Material) found in Table 3; positive ESIMS *m/z*: 425 [M + Na]^+^, HRESIMS *m/z*: 425.1933 (Calculated for: [C_19_H_30_O_9_ + Na]^+^, 425.1928).

*Clausesquiside B* (**9**): ${\left[\alpha \right]}_{D}^{20}$ −101.3° (MeOH; c 0.71); CD (*c* 0.0036, MeOH) Δ*ε* (*λ* nm): −7.1 (321), +186.2 (254) nm; IR (KBr, *ν*_max_, cm^−1^): 3439, 2979, 1675, 1100; NMR data (SI 29–SI 33 in Supplementary Material) found in Table 3; positive ESIMS *m/z*: 409 [M + Na]^+^.

(6R,9R,4Z,7E)-9,10-Dihydroxy-4,7-megastigmadien-3-one-9-*O*-β-d-glucopyranoside (**10**): ${\left[\alpha \right]}_{D}^{20}$: −63.13 (c 0.82, MeOH); ESIMS *m/z*: 393 [M + Na]^+^; ^1^H-NMR (400 MHz, MeOD): *δ* 5.85 (1H, br s, H-4), 5.83 (1H, dd, *J* = 15.4, 9.4 Hz, H-7), 5.66 (1H, dd, *J* = 15.4, 7.4 Hz, H-8), 4.43 (1H, m, H-9), 4.31 (1H, d, *J* = 7.1 Hz, H-1′), 3.90 (1H, dd, 12.0, 2.1 Hz, H-6′a), 3.63 (1H, dd, *J* = 11.0, 3.9 Hz, H-10a), 3.57 (1H, dd, *J* = 11.0, 3.9 Hz, H-10b), 3.54 (1H, m, H-6′b), 3.30–3.13 (4H, m, H-2′, 3′, 4′ and 5′), 2.71 (1H, d, *J* = 9.4 Hz, H-6), 2.43 and 2.00 (1H each, d, *J* = 16.6 Hz, H-2), 1.95 (3H, s, H-13), 1.01 (3H, s, H-12), and 0.97 (3H, s, H-11); ^13^C NMR (101 MHz, MeOD): *δ* 35.7 (s, C-1), 48.3 (t, C-2), 200.6 (s, C-3), 124.9 (d, C-4), 164.1 (s, C-5), 55.6 (d, C-6), 128.9 (d, C-7), 134.1 (d, C-8), 78.3 (d, C-9), 7 (t, C-10), 26.7 (q, C-11), 26.2 (q, C-12), 22.4 (q, C-13), 99.7 (d, C-1′), 3.4 (d, C-2′), 76.7 (d, C-3′), 770.3 (d, C-4′),76.7 (d, C-5′), and 61.4 (d, C-6′) (NMR spectrogram SI 34–SI 35 in Supplementary Material).

*(6R,9S)-Roseoside* (**11**): ${\left[\alpha \right]}_{D}^{20}$: −75.6 (c 1.00, MeOH); ESIMS *m/z*: 409 [M + Na]^+^; ^1^H-NMR (400 MHz, MeOD): δ: 5.90 (1H, d, *J* = 15.6 Hz, H-7), 5.78 (1H, br s, H-4), 5.64 (1H, dd, *J* = 15.6, 7.1 Hz, H-8), 4.44 (1H, m, H-9), 4.18 (1H, d, *J* = 7.5 Hz, H-1′), 3.78 (1H, dd, *J* = 10.5, 2.2 Hz, H- H-6′a), 3.63 (1H, dd, *J* = 10.5, 2.2 Hz, H-6′b), 3.21–3.17 (3H, m, H-2′, 3′ and 4′), 3.11 (1H, m, H-5′), 2.55 (1H, d, *J* = 16.7 Hz, H-2a), 2.16 (1H, d, *J* = 16.7 Hz, H-2b), 1.84 (3H, s, H-13), 1.19 (3H, d, *J* = 6.4 Hz, H-10), 0.94 (3H, s, H-11), and 0.92 (3H, s, H-12); ^13^C NMR (101 MHz, MeOD) *δ*: 41.1 (s, C-1), 49.4 (t, C-2), 200.0 (s, C-3), 125.8 (d, C-4), 165.9 (s, C-5) 78.7 (s, C-6), 132.3 (d, C-7), 132.4 (d, C-8), 73.5 (d, C-9), 23.4 (q, C-10), 20.9 (q, C-11), 22.2 (q, C-12), 18.3 (q, C-13), 99.9 (d, C-1′), 73.3 (d, C-2′), 76.9 (d, C-3′), 70.1 (d, C-4′), 76.8 (d, C-5′), and 61.4 (t, C-6′) (NMR spectrogram SI 36–SI 37 in Supplementary Material).

*Adenosine* (**12**): ESIMS *m/z*: 304 [M + Na]^+^; ^1^H-NMR (400 MHz, MeOD): *δ* 8.07 (1H, s, H-8), 7.90 (1H, s, H-2), 7.13 (2H, br s, 6-NH2), 5.64 (1H, d, *J* = 6.1 Hz, H-1′), 4.38 (1H, m, H-2′), 3.90 (1H, m, H-3′), 3.73 (1H, m, H-4′), 3.41 (1H, m, H-5′a), and 3.35 (1H, m, H-5′b); ^13^C NMR (101 MHz, MeOD) *δ* 153.0 (d, C-2), 149.6 (s, C-4), 119.9 (s, C-5), 156.7 (s, C-6), 140.5 (d, C-8), 88.5 (d, C-1′), 74.1 (d, C-2′), 71.2 (d, C-3′), 86.5 (d, C-4′), and 62.2 (t, C-5′) (NMR spectrogram SI 38–SI 42 in Supplementary Material).

## 3. Materials and Methods

### 3.1. General Experimental Procedures

Optical rotations and UV spectra were measured using a Horiba SEPA-300 polarimeter (Horiba, Tokyo, Japan) and shimadzu UV-2401 A spectrophotometer (Shimadzu, Tokyo, Japan), respectively. IR spectra were recorded with a Tensor 27 Fourier transform infrared spectroscopy (FT-IR) spectrometer with KBr pellets (BioRad, Hercules, CA, USA). Mass Spectrometry (MS) were recorded on an API QSTAR Pular-1 mass spectrometer (VG, Manchester, UK). High Resolution Electrospray Ionization mass Spectroscopy (HRESIMS) were obtained with a Bruker Daltonics, Inc. micro-TOF-Q spectrometer. The ^1^H and ^13^C NMR spectra were acquired with a Bruker AV-400 (^1^H: 400 MHz, ^13^C: 101 MHz) spectrometer in CD_3_OD with tetramethylsilane as the internal standard at room temperature (Bruker, Bremerhaven, Germany). Semipreparative reversed-phase High Performance Liquid Chromatography (HPLC) was performed on an Agilent 1260 apparatus equipped with a UV detector and an Agilent Eclipse (XDB-C18, 5μm, 9.4 × 250 mm) column at a flow rate of 2 mL/min (Agilent, Palo Alto, CA, USA). Column chromatography (CC) was performed on silica gel (100–200 mesh, 200–300 mesh) and TLC was carried out on precoated silica gel GF254 glass plates (Qingdao Marine Chemical, Inc., Qingdao, China). Column chromatography (CC) was performed on sephadex LH-20 (Pharmacia, New Jersey, NJ, USA).

### 3.2. Plant Material

The stems and leaves of *C. lansium* (three-year-old) were collected by pruning and air-dried in Qingyuan (Latitude N 23°70′; Longitude E 113°03′; altitude 71 m), Guangdong Province, China, in September 2015, which were identified by Professor Zhang Zhi-Yong (a botanist) of College of Agriculture, Jiangxi Agricultural University, Nanchang, China. A voucher specimen (no. 2015912) has been deposited in College of Agriculture, Jiangxi Agricultural University.

### 3.3. Extraction and Isolation

The air dried and powdered stems and leaves of *C. lansium* (11 Kg) were extracted by refluxing 95% methanol (20 L each) three times. This process yielded methanol-soluble extracts, which were suspended in water and subsequently extracted with PE, EtOAc, and n-BuOH (3 × 5 L, each), respectively. The n-BuOH part (130 g) was subjected to a reversed-phase column (RP-18) eluting with MeOH-Water (10%–100%) to four sub-fractions (A_1_–A_4_). A_3_ was subjected to normal phase silica gel CC (200–300 mesh) with a gradient system of CH_2_Cl_2_-MeOH (9:1-7:3, v/v) to give six fractions A_3-1_–A_3-6_. A_3-2_ was further separated by normal phase silica gel CC (200–300 mesh) with a isocratic system of CH_2_Cl_2_-MeOH (9:1) to give four fractions A_3-2-1_–A_3-2-4_. A_3-2-2_ was separated by HPLC (mobile phase: H_2_O: MeOH (75:25, v/v)) to give **2** (8 mg), **4** (6 mg), and **5** (9 mg). In the same way, A_3-2-3_ was separated by HPLC [H_2_O: MeOH (80:20, v/v)] to give **1** (6 mg) and **3** (11 mg). Similarly, A_3-2-4_ gave **6** (4 mg) and **7** (10 mg). A_3-3_ was separated by repeated CC (200–300 mesh) with an isocratic mixture of CH_2_Cl_2_-MeOH (9:1, v/v) to produce five fractions A_3-3-1_–A_3-3-5_. After HPLC separation, A_3-3-2_ gave **8** (6 mg) and **10** (9 mg), A_3-3-3_ gave **9** (5 mg), and A_3-3-4_ produced **11** (8 mg) and **12** (13 mg) [35,36,48].

### 3.4. Determination of Absolute Configurations of Sugars

Compounds **1**,**6,8**,**9** (each compound 2–3 mg) were dissolved in 1 N HCl (4 mL) and heated at 90 °C under condition of reflux for 6 h. The reaction product was dissolved in H_2_O after evaporation and partitioned with CH_2_Cl_2_. The aqueous layer containing sugars was concentrated, and then was mixed with L-cysteine methyl ester hydrochloride. Anhydrous pyridine (1 mL) was added to the mixture and heated at 60 °C for 2 h. The product was added to isothiocyanate (3 mg) and heated at 60 °C for another 2 h. The final reaction mixture was analyzed by HPLC under the following conditions: an Agilent 1260 chromatograph equipped an Eclipse XDB-C18 column (5 µm, 4.6 × 250 mm); column temperature: 35 °C; mobile phase: isocratic elution of 25% CH_3_CN–H_2_O (V:V) in 50 mmol/L HCl; flow rate: 0.8 mL/min; injection volume: 10 µL; and UV detection wavelength: 250 nm. The standard d-glucose and d-apiose were subjected under the same conditions. After the comparison of the retention times of monosaccharide derivatives, the samples were confirmed to comprise of d-glucose (19.25 min) and d-apiose (30.34 min), respectively [19,20].

## 4. Conclusions

In conclusion, 12 compounds were isolated from *C. lansium*. They were obtained from the genus *Clausena* for the first time, including four new glycosides. The existence of these compounds demonstrates the taxonomic significance of *C. lansium* in the genus *Clausena* and suggests that some glycosides from this plant probably play a role in the anticancer activity of *C. lansium* to some extent.

## Figures and Tables

**Table 1:** ^1^H NMR (400 MHz, MeOD) and ^13^C NMR (101 MHz, MeOD) spectroscopic data of **1**.

Position	*δ*_H_ (*J* in Hz)	*δ*_C_	Position	*δ*_H_ (*J* in Hz)	*δ*_C_
1		137.6 (s)	4′	3.84 (m)	76.6 (d)
2	7.31 (d, 7.2)	127.8 (d)	5′	3.51 (m)	77.2 (d)
3	7.23 (m)	127.9 (d)	6′	3.78 (m)	61.4 (t)
4	7.18 (d, 7.3)	127.3 (d)		3.60 (m)	
5	7.23 (m)	127.9 (d)	1″	5.27 (d, 2.1)	109.3 (d)
6	7.31 (d, 7.2)	127.8 (d)	2″	3.31 (m)	77.6 (d)
7	4.56 (d,11.6)	70.4 (t)	3″		79.2 (s)
	4.80 (d,11.6)		4″	3.81 (m)	73.9 (t)
1′	4.30 (d,7.5)	100.8 (d)		3.55 (m)	
2′	3.16 (m)	76.5 (d)	5″	3.45 (d, 12.2)	64.6 (t)
3′	3.38 (m)	70.3 (d)		3.41 (d, 12.2)	

**Table 2:** ^1^H NMR (400 MHz, MeOD) and ^13^C NMR (101 MHz, MeOD) spectroscopic data of **6**.

Position	*δ*_H_ (*J* in Hz)	*δ*_C_	Position	*δ*_H_ (*J* in Hz)	*δ*_C_
2		161.3 (s)	1″		77.5 (s)
3	6.25 (d, 9.5)	110.7 (d)	2″	1.64 (3H, s)	23.3 (q)
4	7.91 (d, 9.5)	144.9 (d)	3″	1.63 (3H, s)	22.2 (q)
4a		113.8 (s)	1‴	4.85 (d, 7.5)	97.2 (d)
5	7.21 (s)	114.3 (d)	2‴	3.16 (m)	73.1 (d)
6		127.8 (s)	3‴	3.41 (m)	76.1 (d)
7		150.1 (s)	4‴	3.40 (m)	69.3 (d)
8		129.3 (s)	5‴	3.19 (m)	75.6 (d)
8a		144.1 (s)	6‴	3.50 (m)	59.9 (t)
2′	4.57 (d, 6.5)	91.5 (d)		3.17 (m)	
3′	5.35 (d, 6.5)	71.1 (d)			

**Table 3:** ^1^H NMR (400 MHz, MeOD) and ^13^C NMR (101 MHz, MeOD) spectroscopic data of **8** and **9**.

Position	8		Position	9	
	*δ*_H_ (*J* in Hz)	*δ*_C_		*δ*_H_ (*J* inHz)	*δ*_C_
1		40.5 (s)	1		36.8 (s)
2	2.61 (d, 16.6)	48.8 (t)	2	2.48 (d, 15.8)	48.4 (t)
	2.16 (d, 16.6)			2.11 (d, 15.8)	
3		199.5 (s)	3		200.8 (s)
4	5.85 (s)	125.3 (d)	4	5.79 (br s)	124.9 (d)
5		165.3 (s)	5		164.3 (s)
6		78.4 (s)	6	2.72 (d, 9.8)	57.1 (d)
7	6.92 (dd, 15.6, 6.3)	127.0 (d)	7	5.82 (dd, 15.4, 9.8)	130.7 (d)
8	5.79 (dd, 15.6, 7.4)	133.9 (d)	8	5.55 (dd, 15.4, 7.1)	132.1 (d)
9	4.45 (m)	77.6 (d)	9	4.30 (m)	78.2 (d)
10	3.60 (dd, 11.8, 4.2)	64.1 (d)	10	3.61 (dd, 11.6, 4.1)	64.6 (t)
	3.57 (dd, 11.8, 4.2)			3.57 (dd, 11.6, 4.1)	
11	0.95 (3H, s),	21.6 (q)	11	0.93 (3H, s),	26.7 (q)
12	0.93 (3H, s),	22.9 (q)	12	0.88 (3H, s),	26.2 (q)
13	1.91 (3H, s),	17.7 (q)	13	2.04 (3H, s),	22.6 (q)
1′	4.26 (d, 7.6)	99.5 (d)	1′	4.21 (d, 7.3)	99.9 (d)
2′	3.25 (m)	73.0 (d)	2′	3.32 (m)	73.5 (d)
3′	3.24 (m)	76.2 (d)	3′	3.25 (m)	76.7 (d)
4′	3.23 (m)	69.6 (d)	4′	3.22 (m)	70.2 (d)
5′	3.13 (m)	76.2 (d)	5′	3.14 (m)	76.7 (d)
6′	3.81 (dd, 11.2, 2.1)	60.8 (t)	6′	3.73 (dd, 12.1, 2.2)	61.3 (d)
	3.64 (dd, 11.2, 2.1)			3.68 (dd, 12.1, 2.2)	

## Supplementary Materials

The 1D- and 2D-NMR spectroscopic data of compounds **1**–**12** in the paper are available online.
