# Supplementary material for: Phytochemical Study of Stem and Leaf of Clausena lansium
Source: Molecules. 2019 Aug 28;24(17):3124. doi: 10.3390/molecules24173124 (PMC6749519; doi:10.3390/molecules24173124)
Supplement: Supplementary file 1 [file molecules-24-03124-s001.pdf]

Supplementary Information

Phytochemical Study of Stem and Leaf of *Clausena lansium*

Wenwen Peng[1,2*], Xiaoxiang Fu[2], Yuyan Li[2], Zhonghua Xiong[2], Xugen Shi[2], Fang Zhang[2], and Baotong Li[3*]

[1] Jiangxi Key Laboratory for Conservation and Utilization of Fungal Resources, Jiangxi Agricultural University, Nanchang 330045, China; pengwenwen123@sina.com (W.W.P.);

[2] College of Agriculture, Jiangxi Agricultural University, Nanchang 330045, China; xiaoxiangfu@jxau.edu.cn (X.X.F.); 845217104@qq.com (Y.Y.L.); 63701313@qq.com (Z.H.X.); 275507215@qq.com (X.G.S.)

[3] School of Land Resources and Environment，Jiangxi Agricultural University，Nanchang 330045, P.R. China; e-mail: libt66@163.com (B.T.L.);

* Correspondence: pengwenwen123@sina.com (W.W.P.); libt66@163.com (B.T.L.).

Contents

SI 1: ^{1}H-NMR spectrum of compound **1**.

SI 2: ^{13}C-NMR and DEPT spectrum of compound **1**.

SI 3: HSQC spectrum of compound **1**.

SI 4: ^{1}H-^{1}H COSY spectrum of compound **1**.

SI 5: HMBC spectrum of compound **1**

SI 6: ^{1}H-NMR spectrum of compound **2**.

SI 7: ^{13}C-NMR and DEPT spectrum of compound **2**.

SI 8: ^{1}H-NMR spectrum of compound **3**.

SI 9: ^{13}C-NMR and DEPT spectrum of compound **3**.

SI 10: ^{1}H-NMR spectrum of compound **4**.

SI 11: ^{13}C-NMR and DEPT spectrum of compound **4**.

SI 12: HSQC spectrum of compound **4**.

SI 13: ^{1}H-^{1}H COSY spectrum of compound **4**.

SI 14: HMBC spectrum of compound **4**

SI 15: ^{1}H-NMR spectrum of compound **5**.

SI 16: ^{13}C-NMR and DEPT spectrum of compound **5**.

SI 17: ^{1}H-NMR spectrum of compound **6**.

SI 18: ^{13}C-NMR and DEPT spectrum of compound **6**.

SI 19: HSQC spectrum of compound **6**.

SI 20: ^{1}H-^{1}H COSY spectrum of compound **6**.

SI 21: HMBC spectrum of compound **6**

SI 22: ^{1}H-NMR spectrum of compound **7**.

SI 23: ^{13}C-NMR and DEPT spectrum of compound **7**.

SI 24: HSQC spectrum of compound **7**.

SI 25: ^{1}H-^{1}H COSY spectrum of compound **7**.

SI 26: HMBC spectrum of compound **7**

SI 27: ^{1}H-NMR spectrum of compound **8**.

SI 28: ^{13}C-NMR and DEPT spectrum of compound **8**.

SI 29: ^{1}H-NMR spectrum of compound **9**.

SI 30: ^{13}C-NMR and DEPT spectrum of compound **9**.

SI 31: HSQC spectrum of compound **9**.

SI 32: ^{1}H-^{1}H COSY spectrum of compound **9**.

SI 33: HMBC spectrum of compound **9.**

SI 34: ^{1}H-NMR spectrum of compound **10**.

SI 35: ^{13}C-NMR and DEPT spectrum of compound **10**.

SI 36: ^{1}H-NMR spectrum of compound **11**.

SI 37: ^{13}C-NMR and DEPT spectrum of compound **11**.

SI 38: ^{1}H-NMR spectrum of compound **12**.

SI 39: ^{13}C-NMR and DEPT spectrum of compound **12**.

SI 40: HSQC spectrum of compound **12**.

SI 41: ^{1}H-^{1}H COSY spectrum of compound **12**.

SI 42: HMBC spectrum of compound **12.**

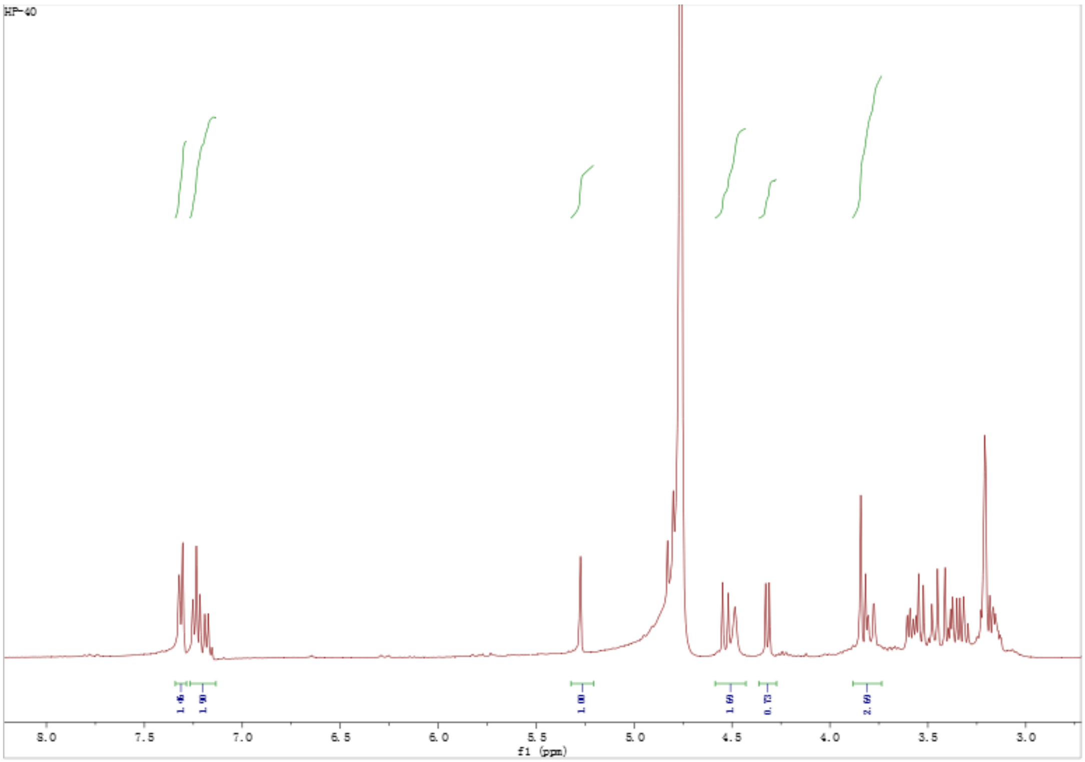

SI 1: ^{1}H-NMR spectrum of compound **1**.

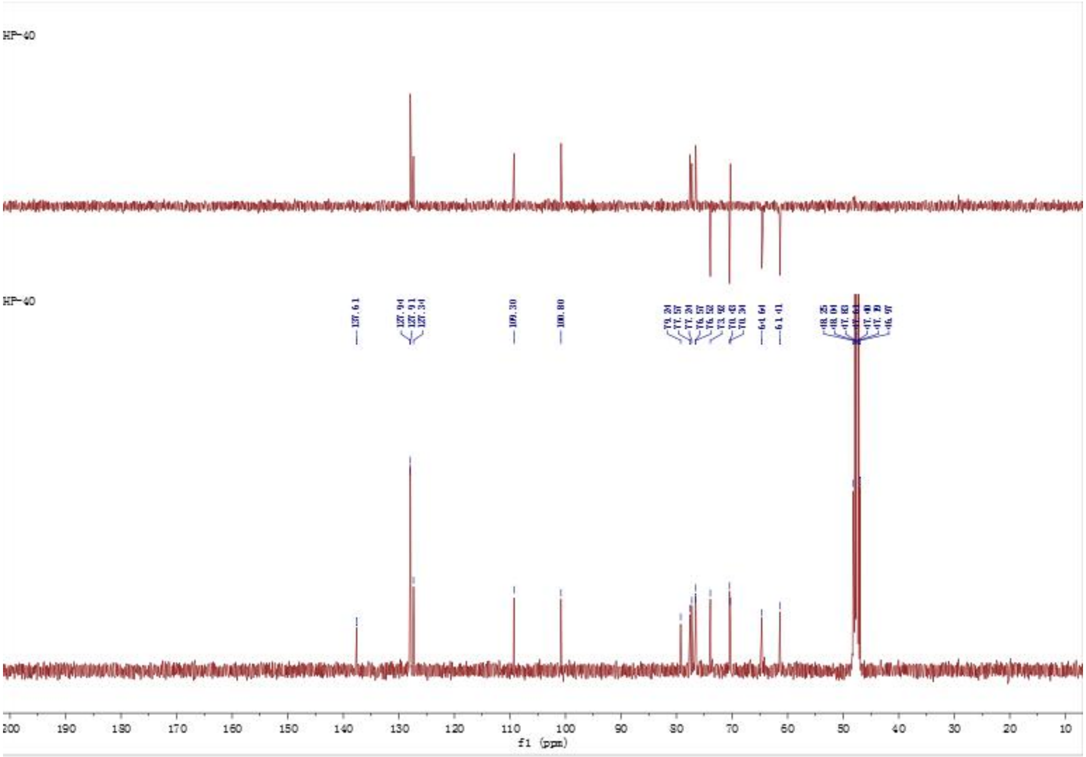

SI 2: ^{13}C-NMR and DEPT spectrum of compound **1**.

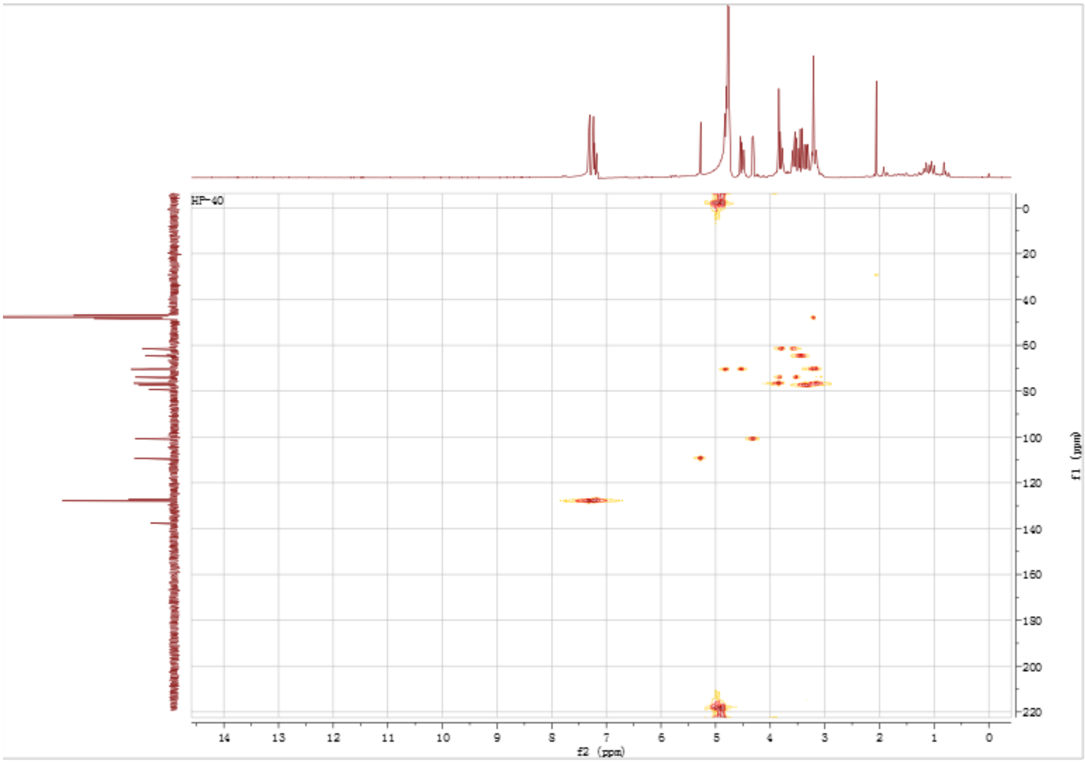

SI 3: HSQC spectrum of compound **1**.

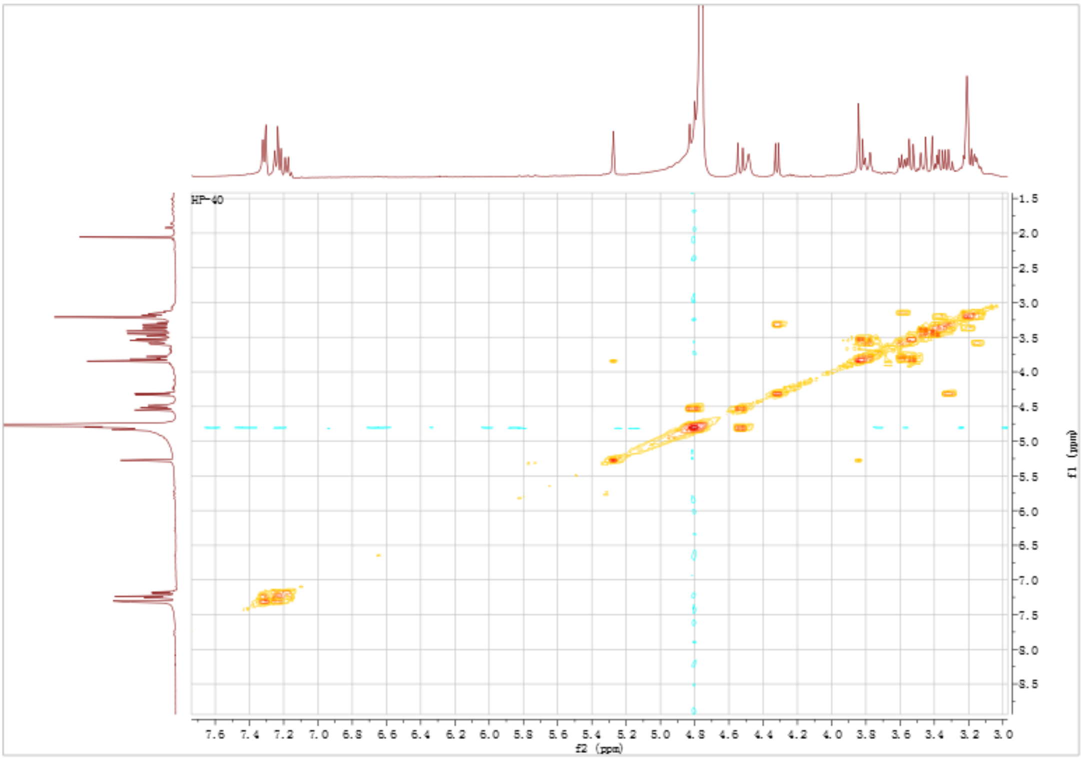

SI 4: ^{1}H-^{1}H COSY spectrum of compound **1**.

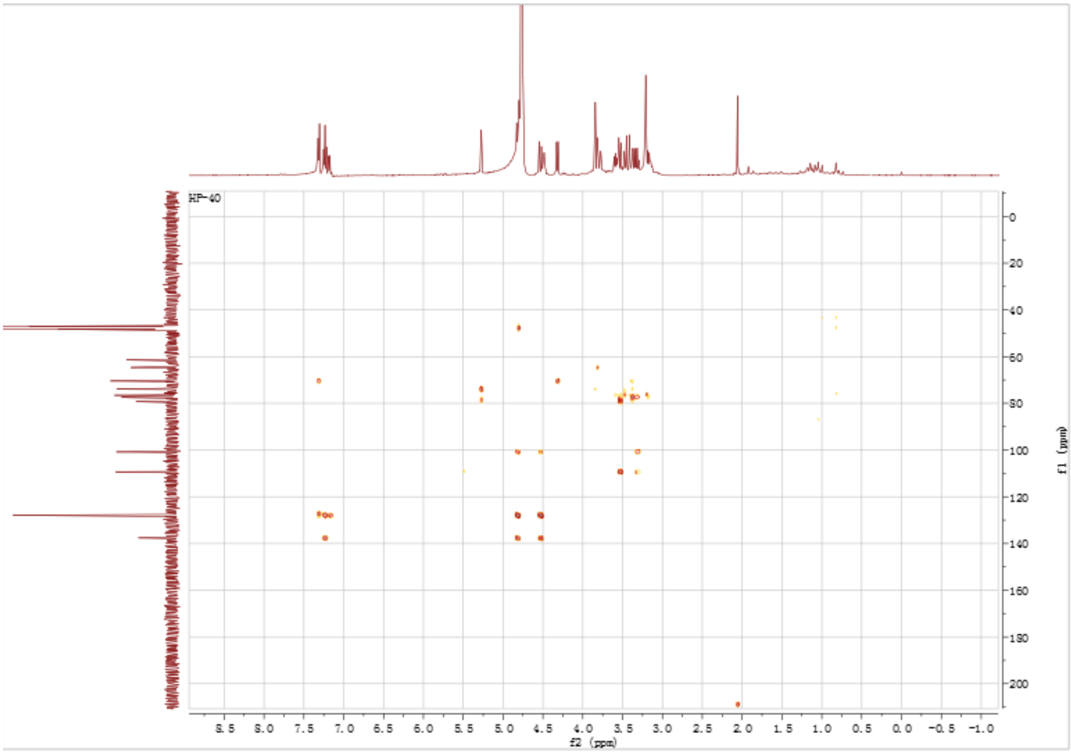

SI 5: HMBC spectrum of compound **1**

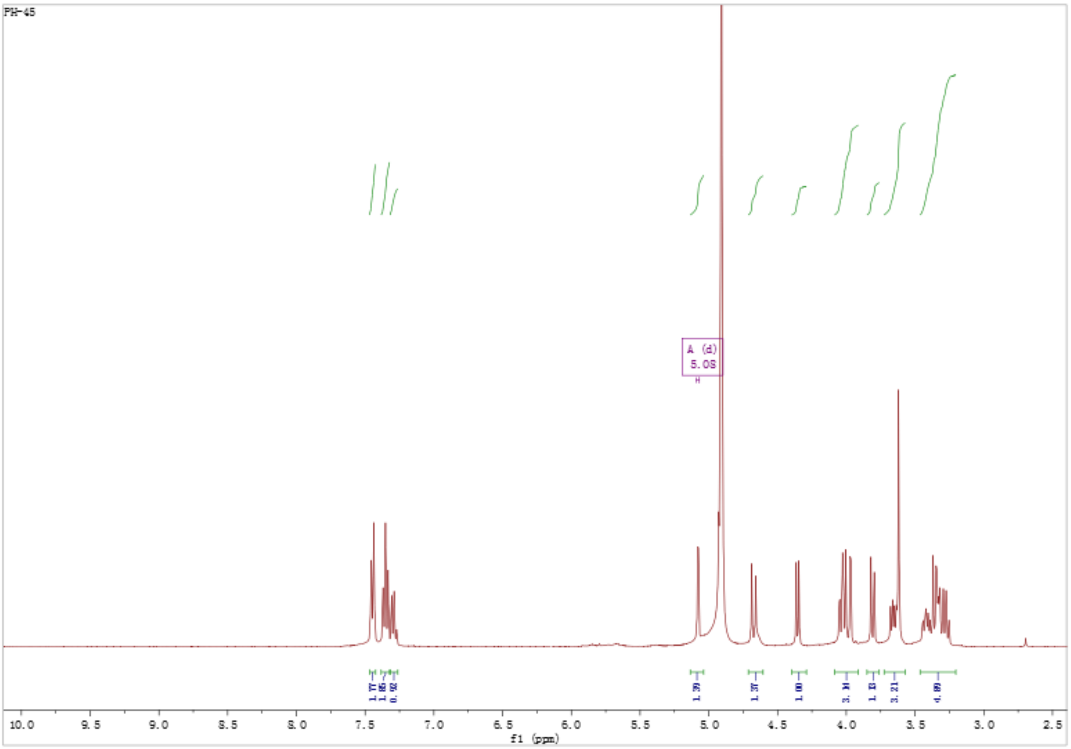

SI 6: ^{1}H-NMR spectrum of compound **2**.

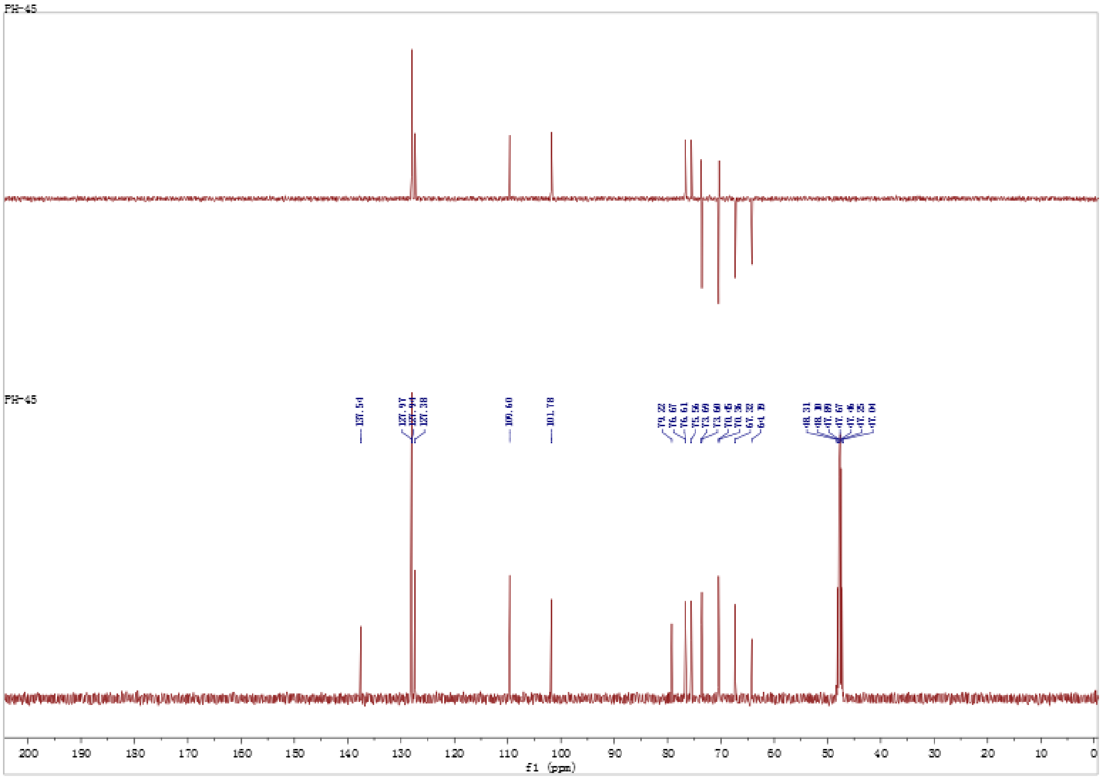

SI 7: ^{13}C-NMR and DEPT spectrum of compound **2**.

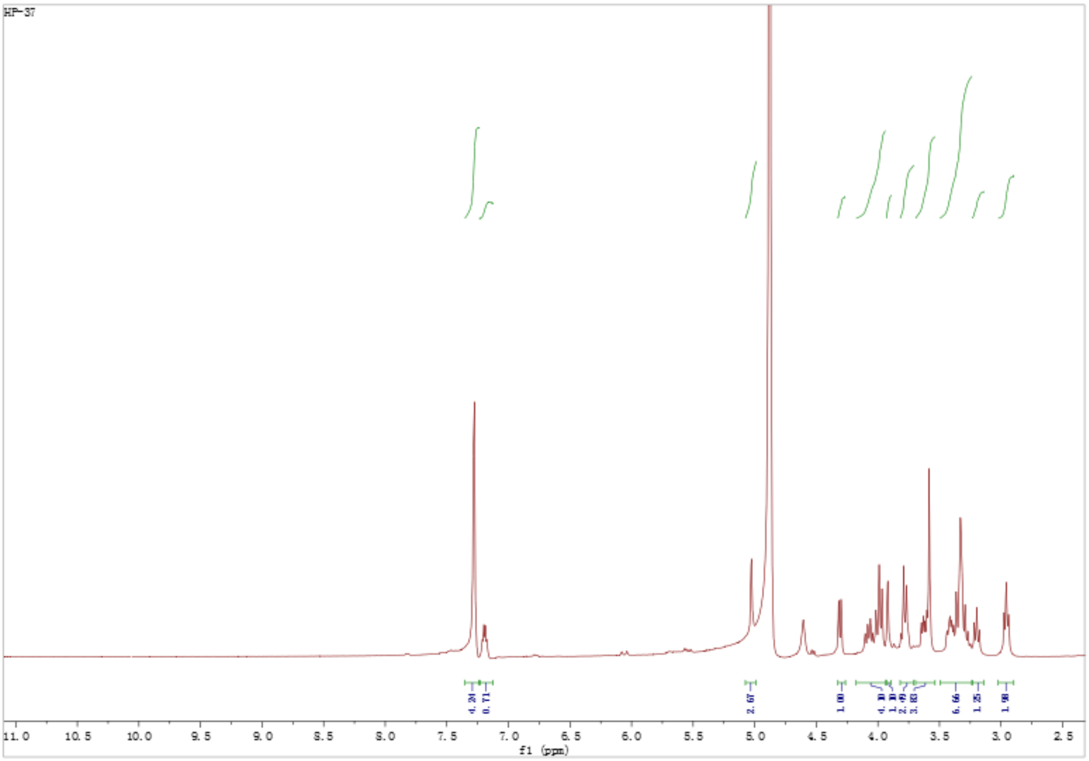

SI 8: ^{1}H-NMR spectrum of compound **3**.

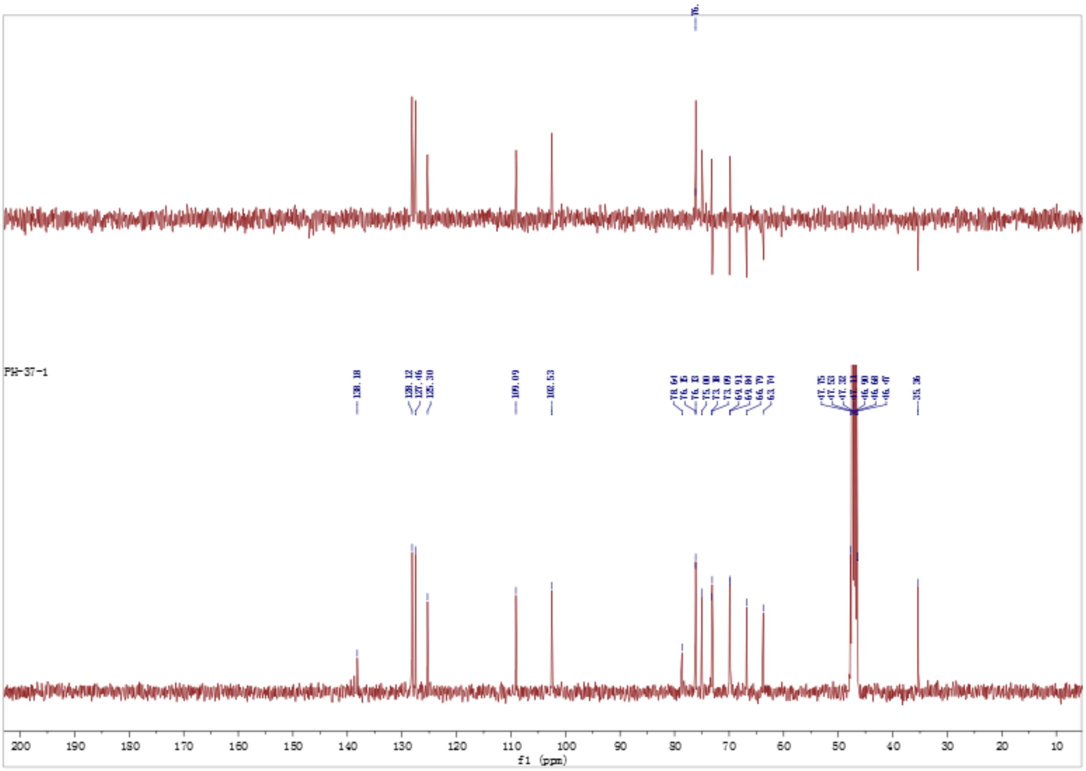

SI 9: ^{13}C-NMR and DEPT spectrum of compound **3**.

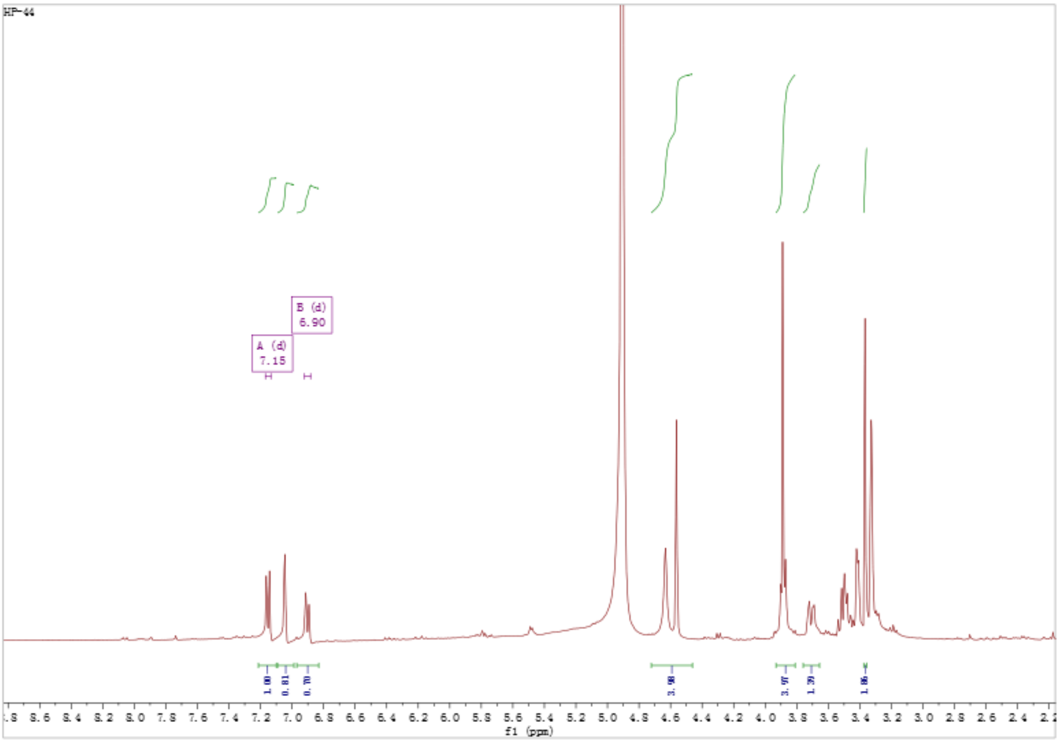

SI 10: ^{1}H-NMR spectrum of compound **4**.

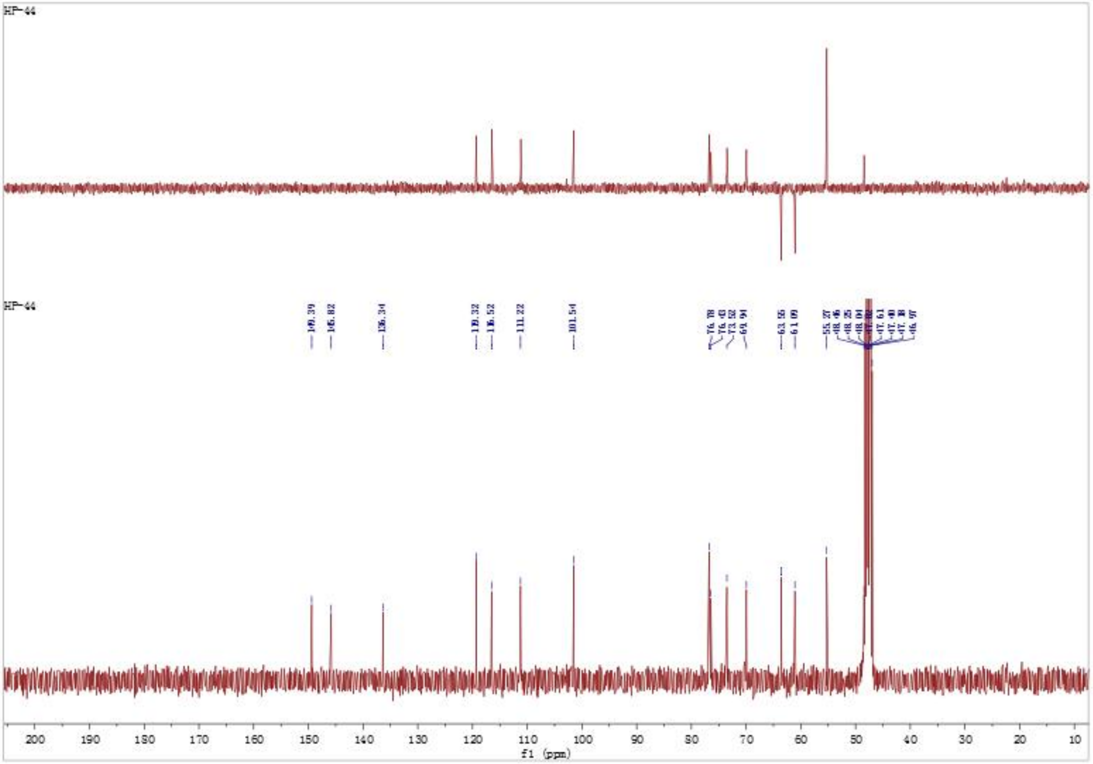

SI 11: ^{13}C-NMR and DEPT spectrum of compound **4**.

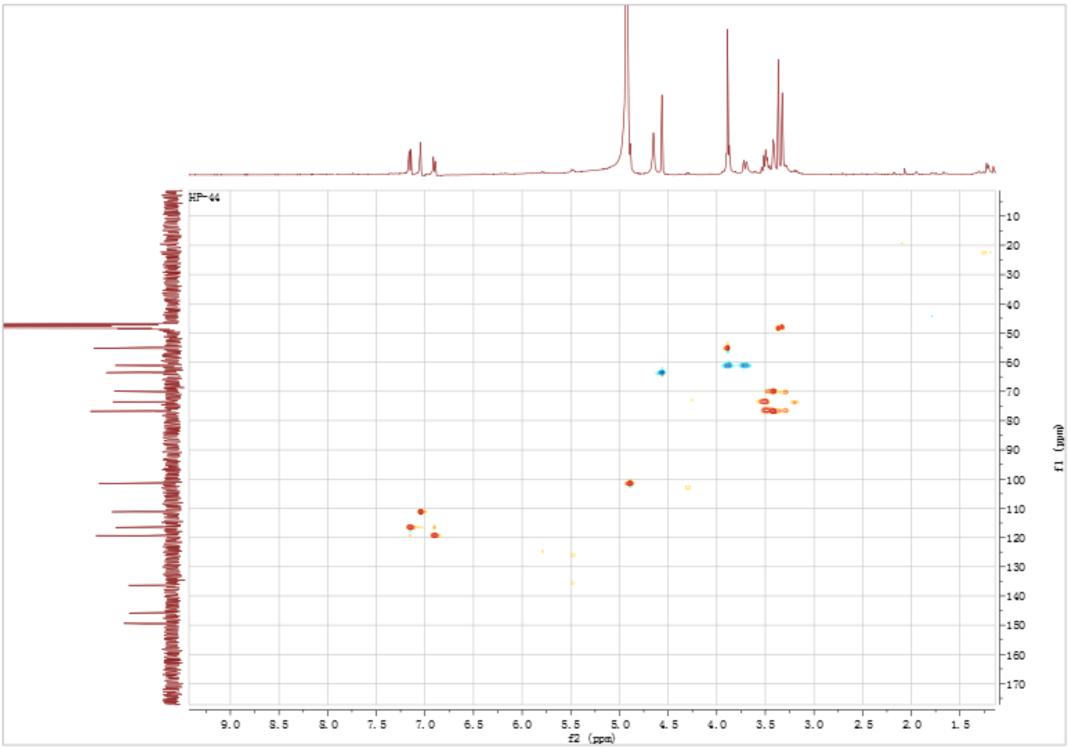

SI 12: HSQC spectrum of compound **4**.

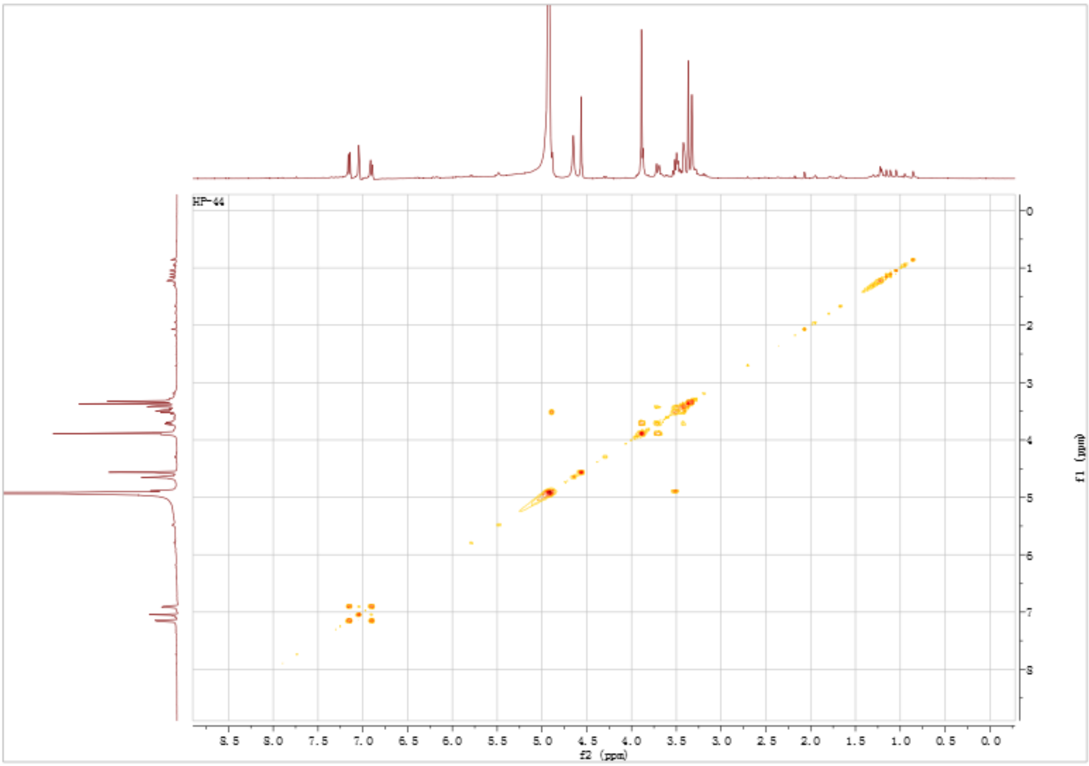

SI 13: ^{1}H-^{1}H COSY spectrum of compound **4.**

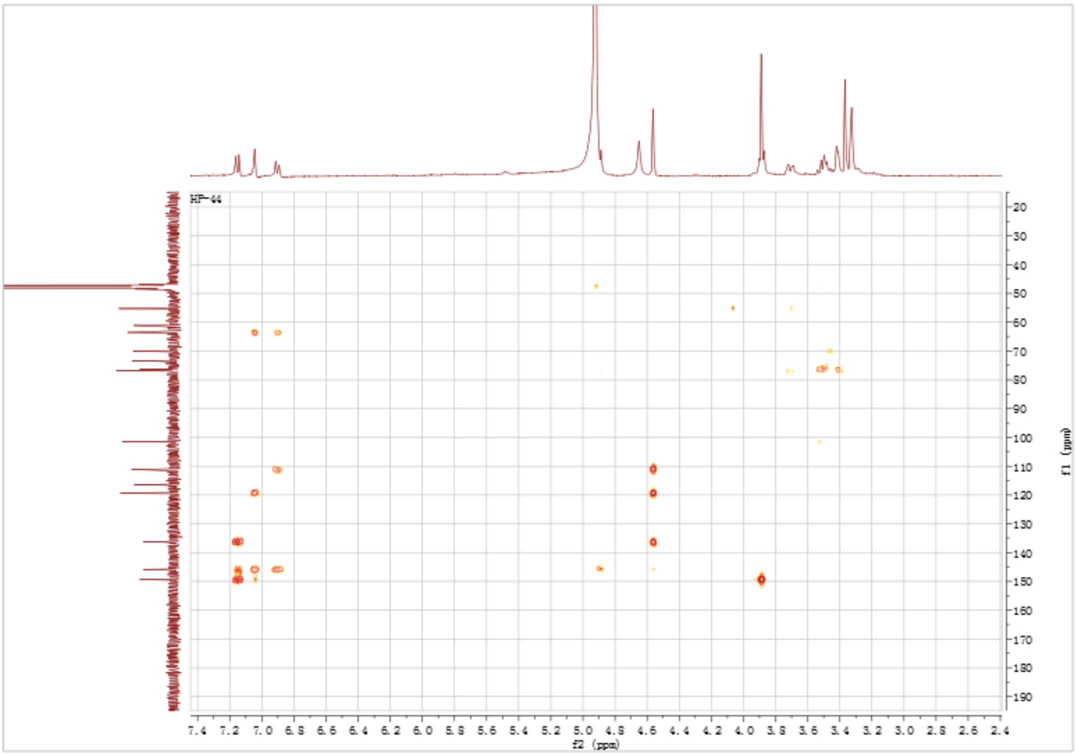

SI 14: HMBC spectrum of compound **4**

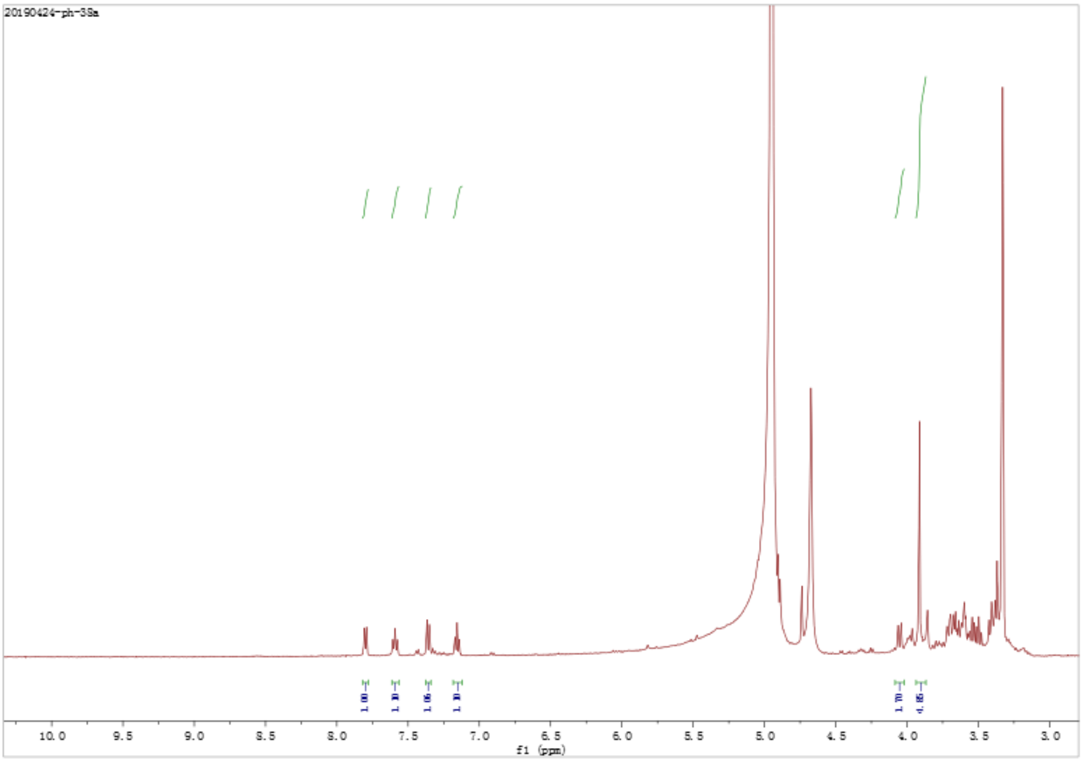

SI 15: ^{1}H-NMR spectrum of compound **5**.

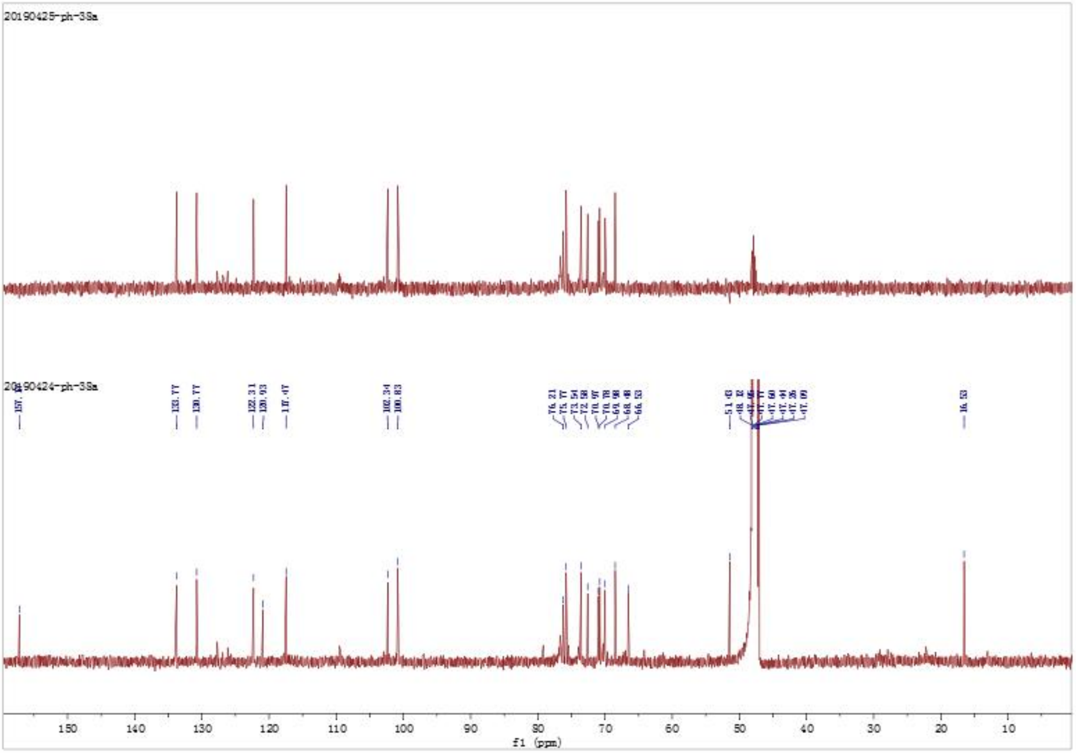

SI 16: ^{13}C-NMR and DEPT spectrum of compound **5**.

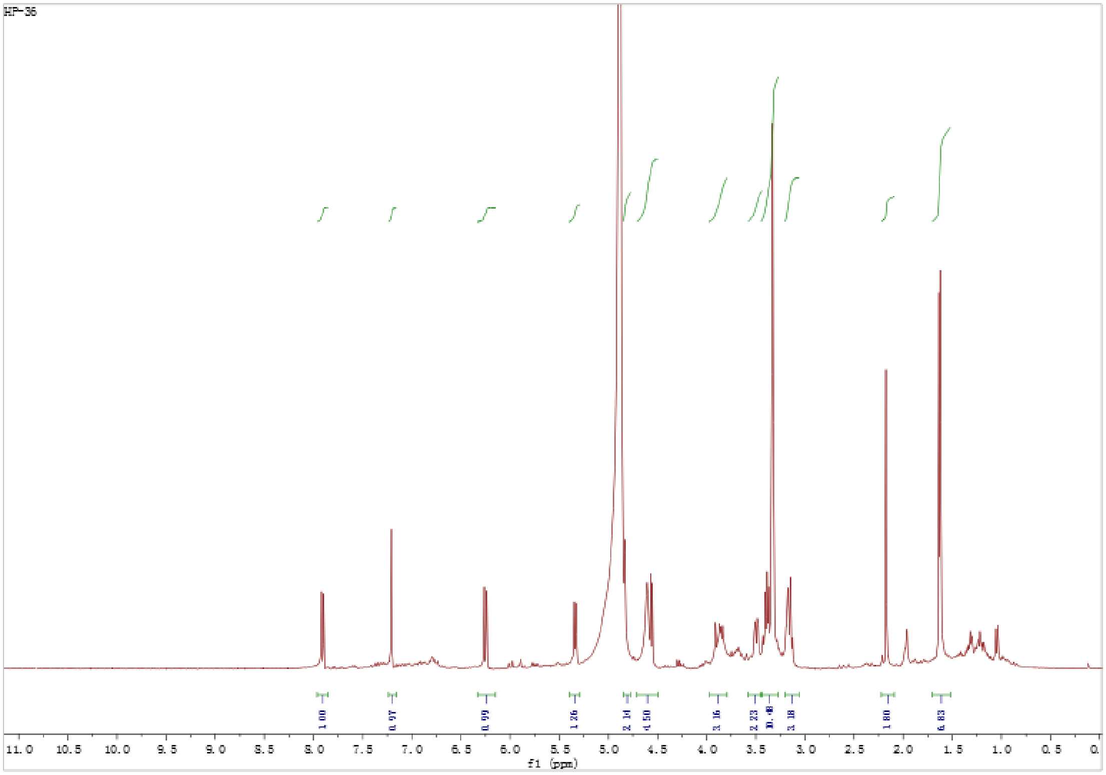

SI 17: ^{1}H-NMR spectrum of compound **6**.

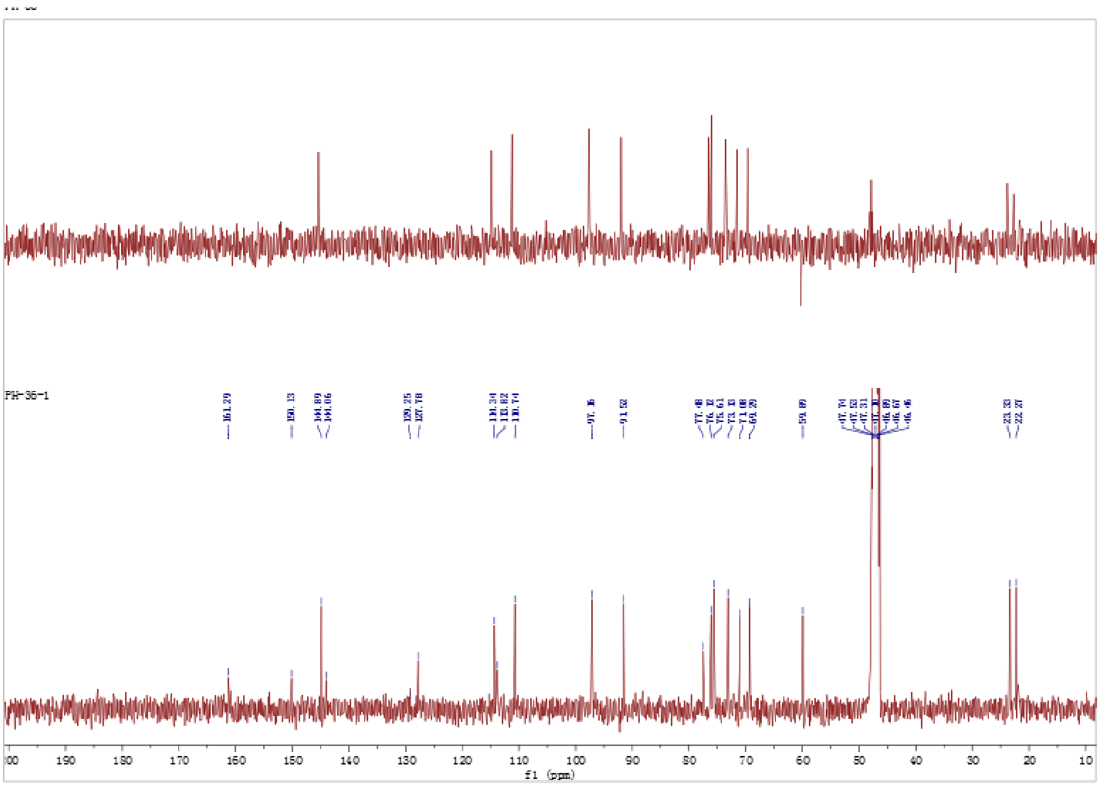

SI 18: ^{13}C-NMR and DEPT spectrum of compound **6**.

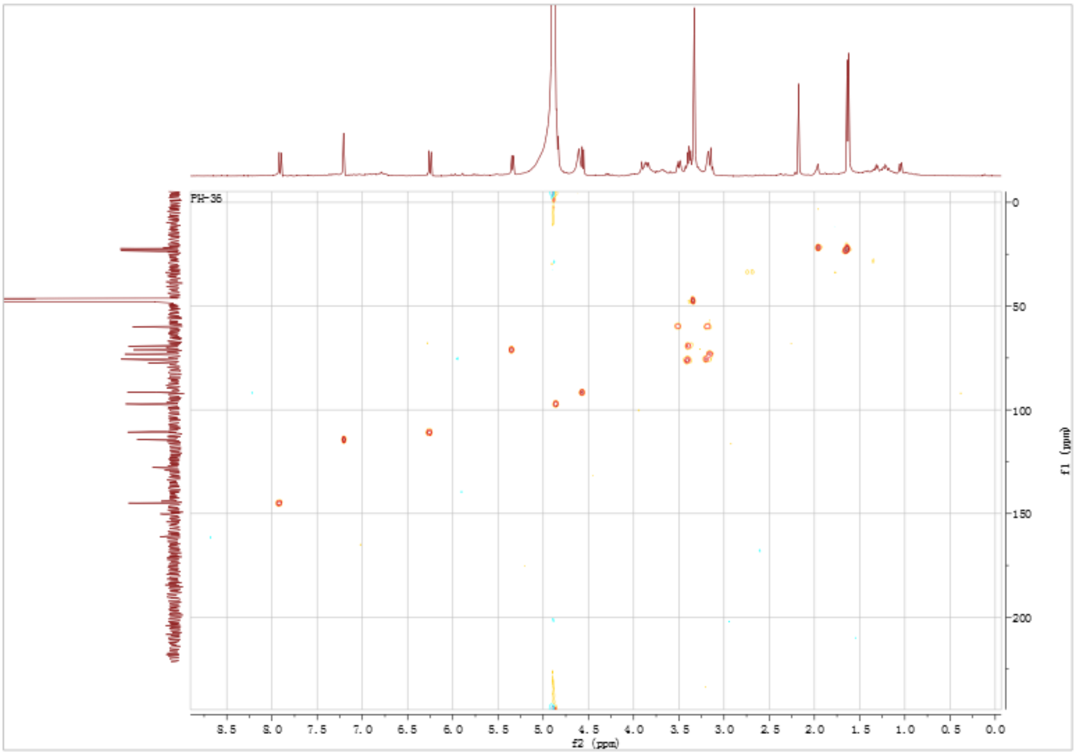

SI 19: HSQC spectrum of compound **6**.

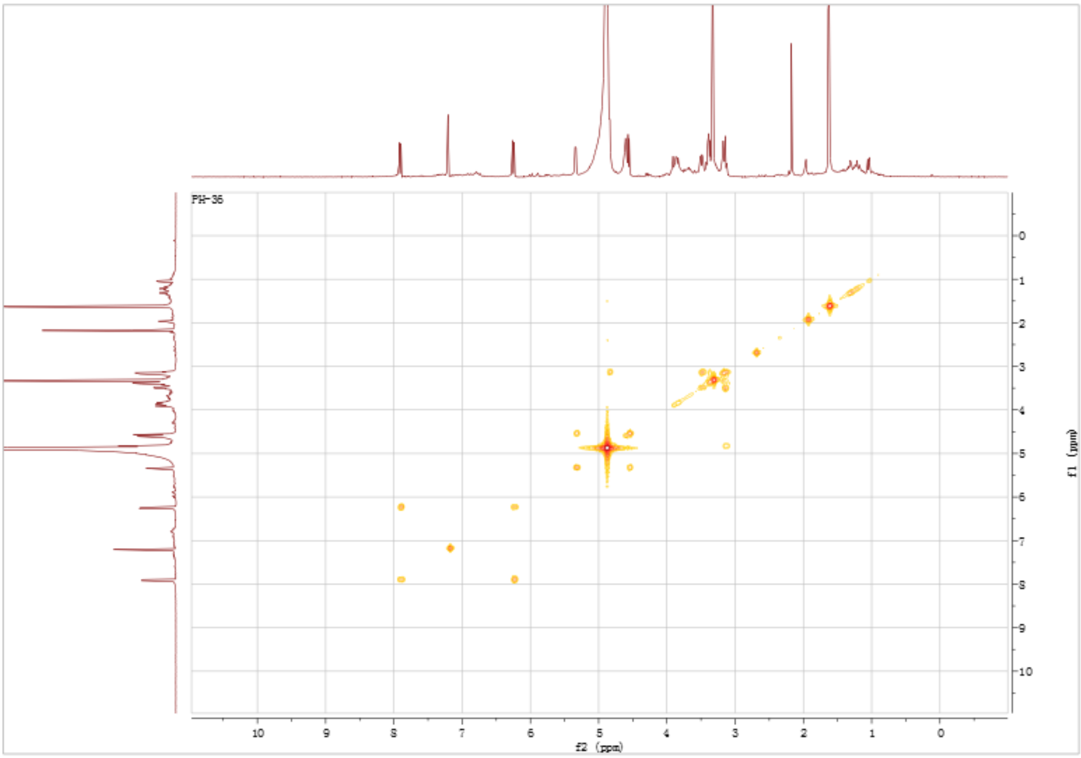

SI 20: ^{1}H-^{1}H COSY spectrum of compound **6**.

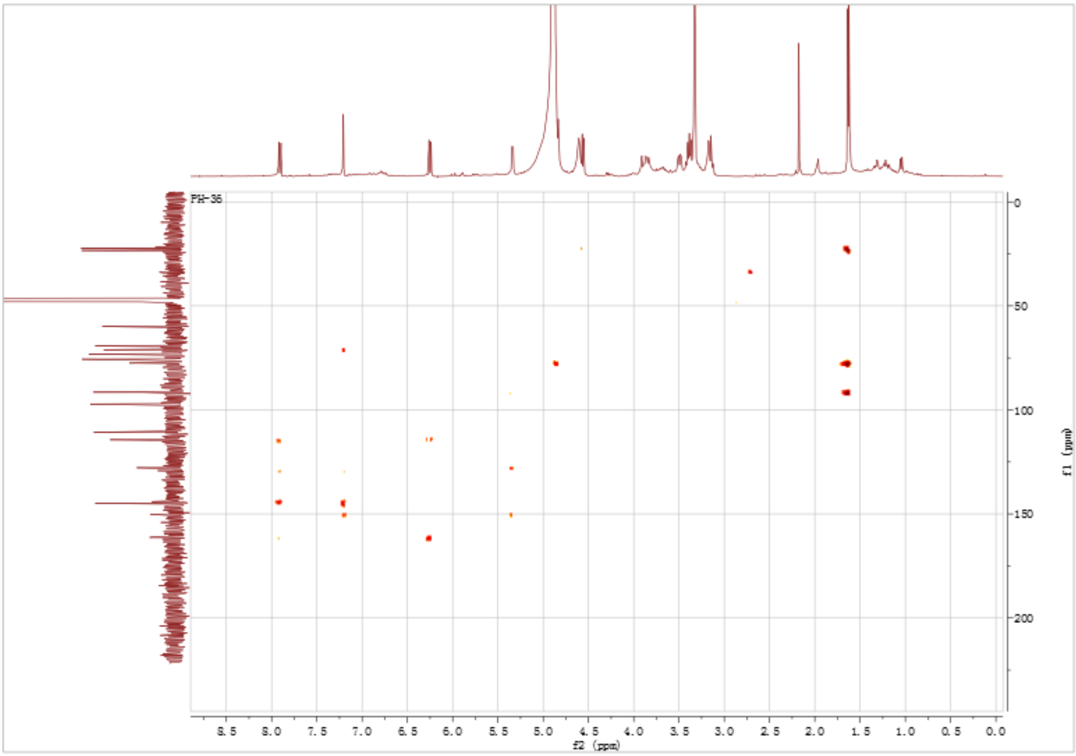

SI 21: HMBC spectrum of compound **6**

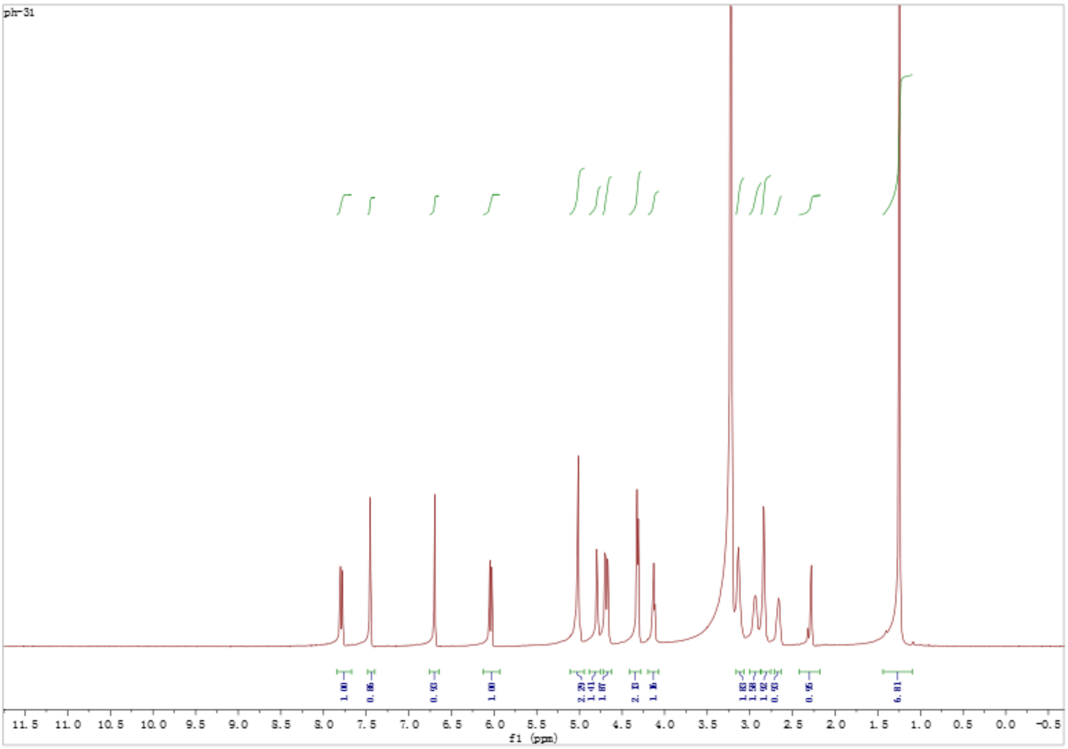

SI 22: ^{1}H-NMR spectrum of compound **7**.

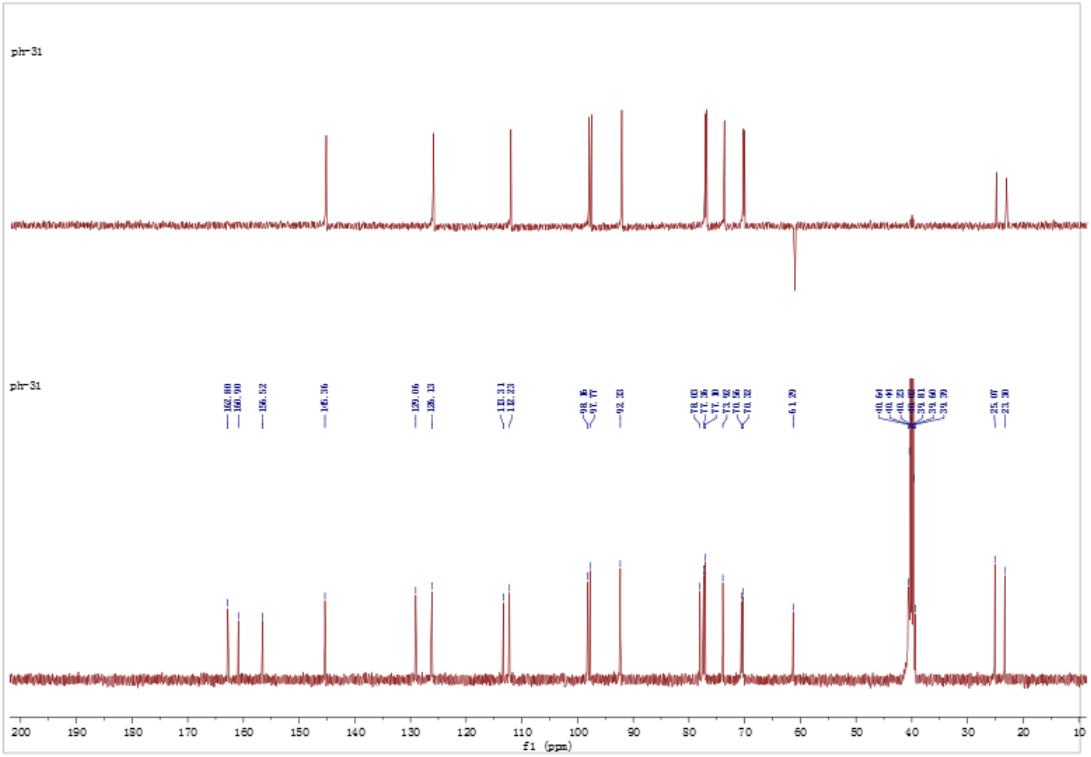

SI 23: ^{13}C-NMR and DEPT spectrum of compound **7**.

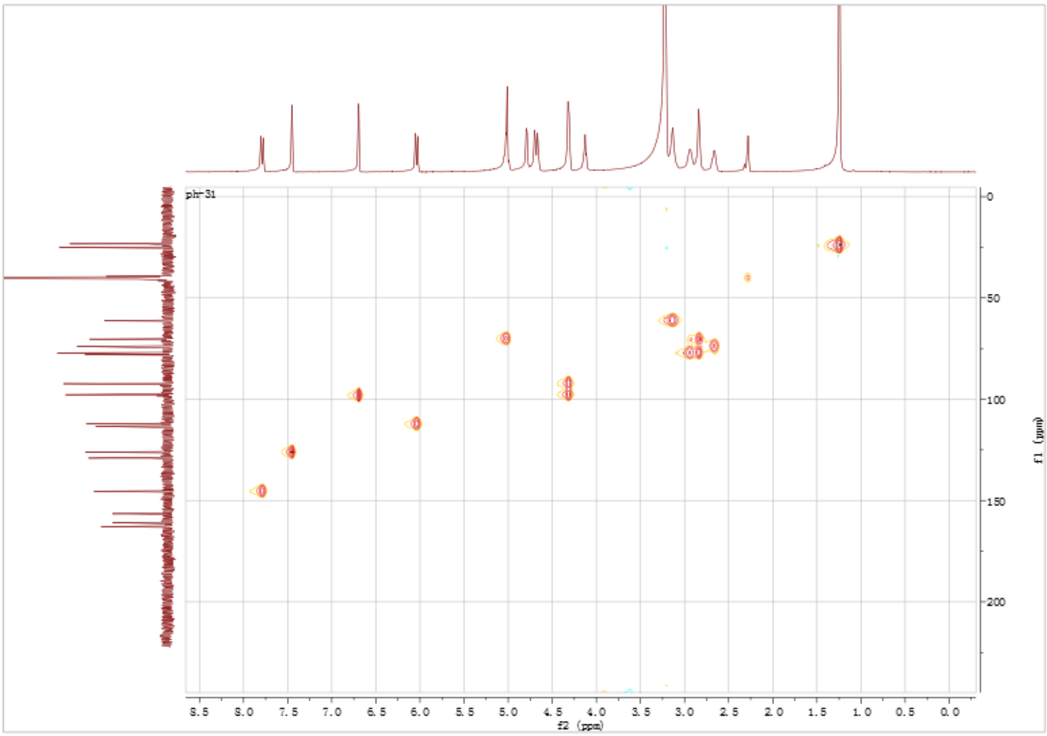

SI 24: HSQC spectrum of compound **7**.

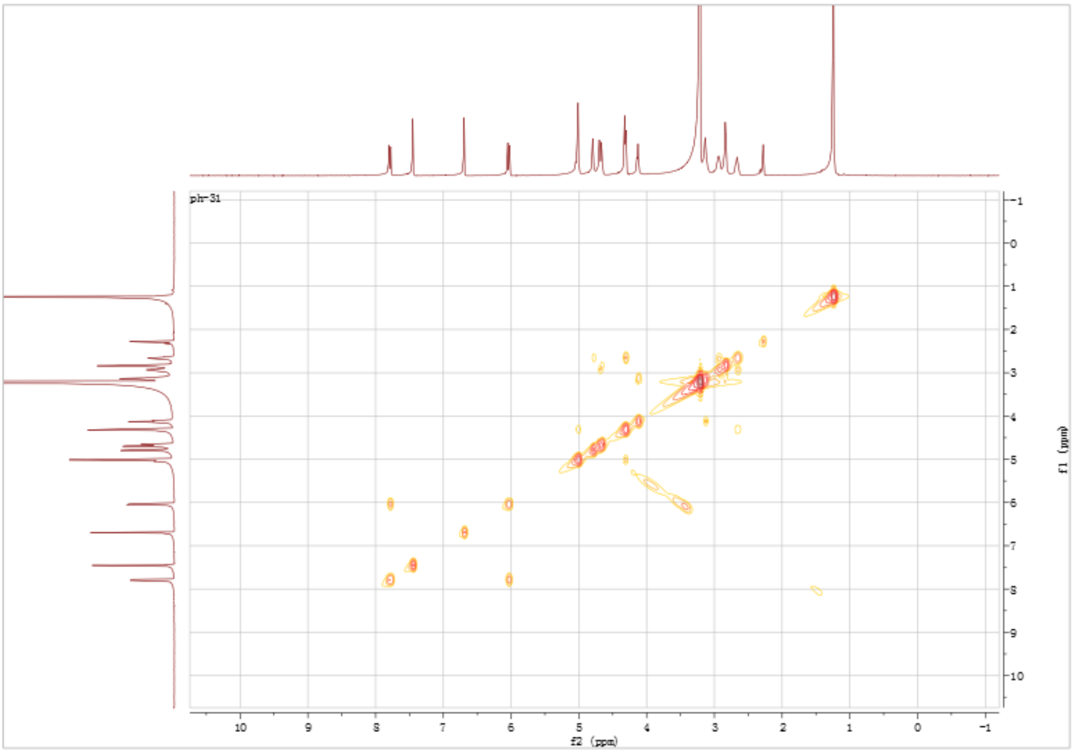

SI 25: ^{1}H-^{1}H COSY spectrum of compound **7**.

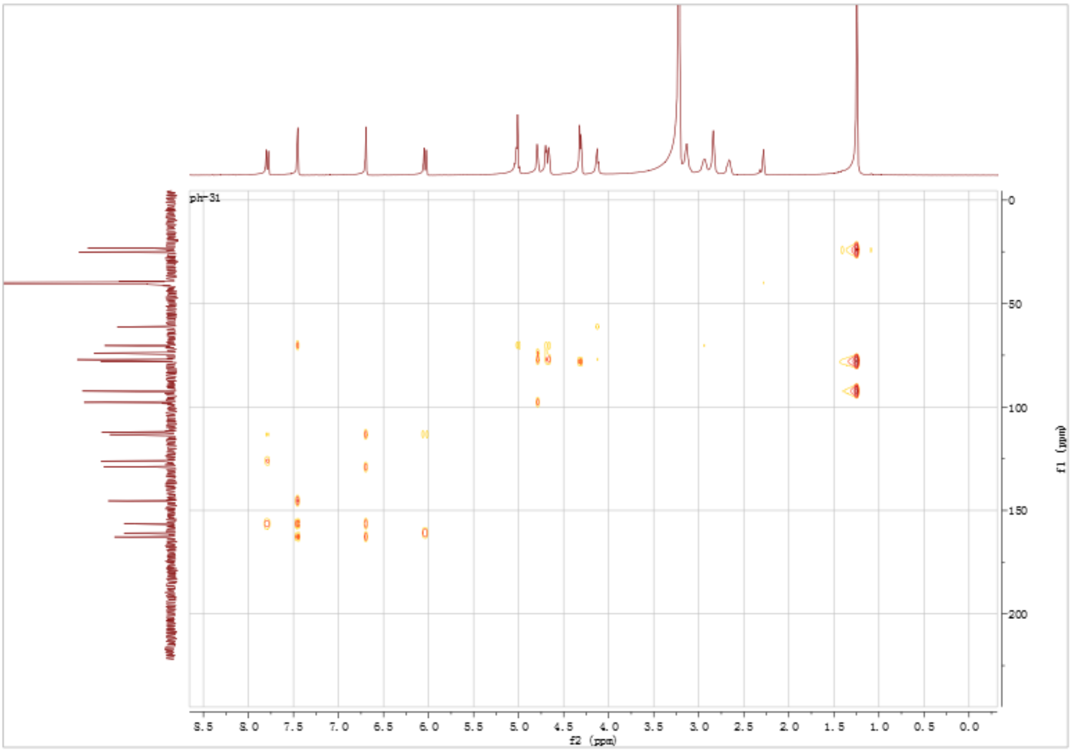

SI 26: HMBC spectrum of compound **7**

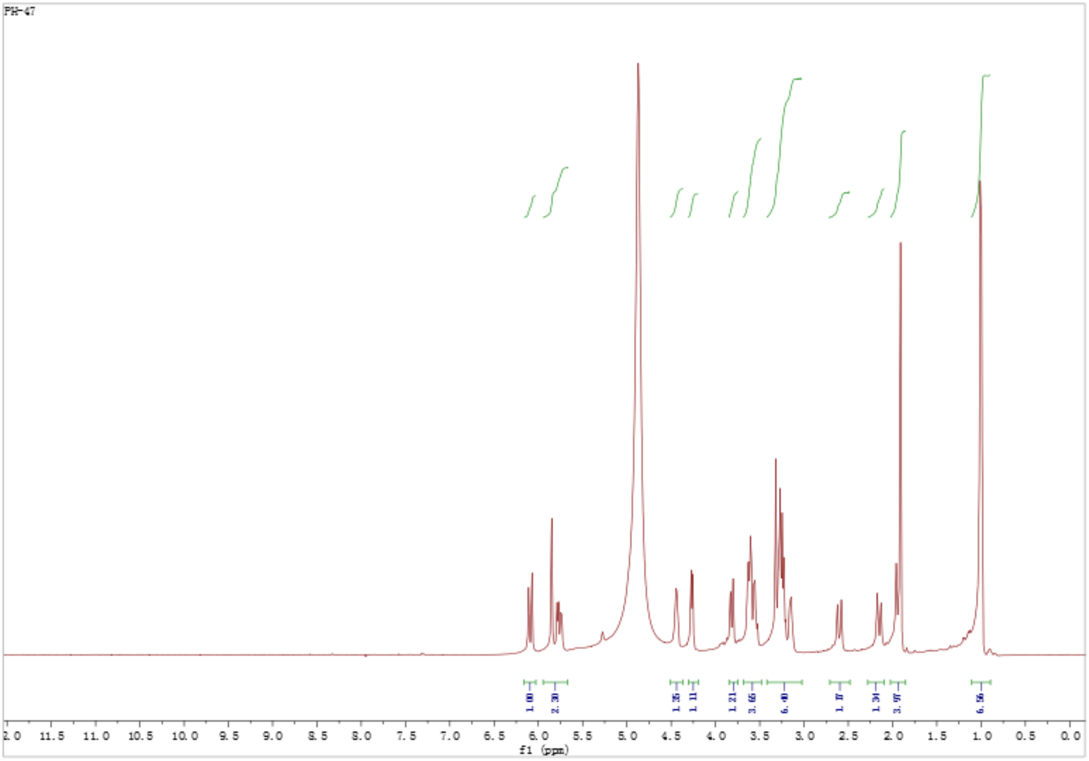

SI 27: ^{1}H-NMR spectrum of compound **8**.

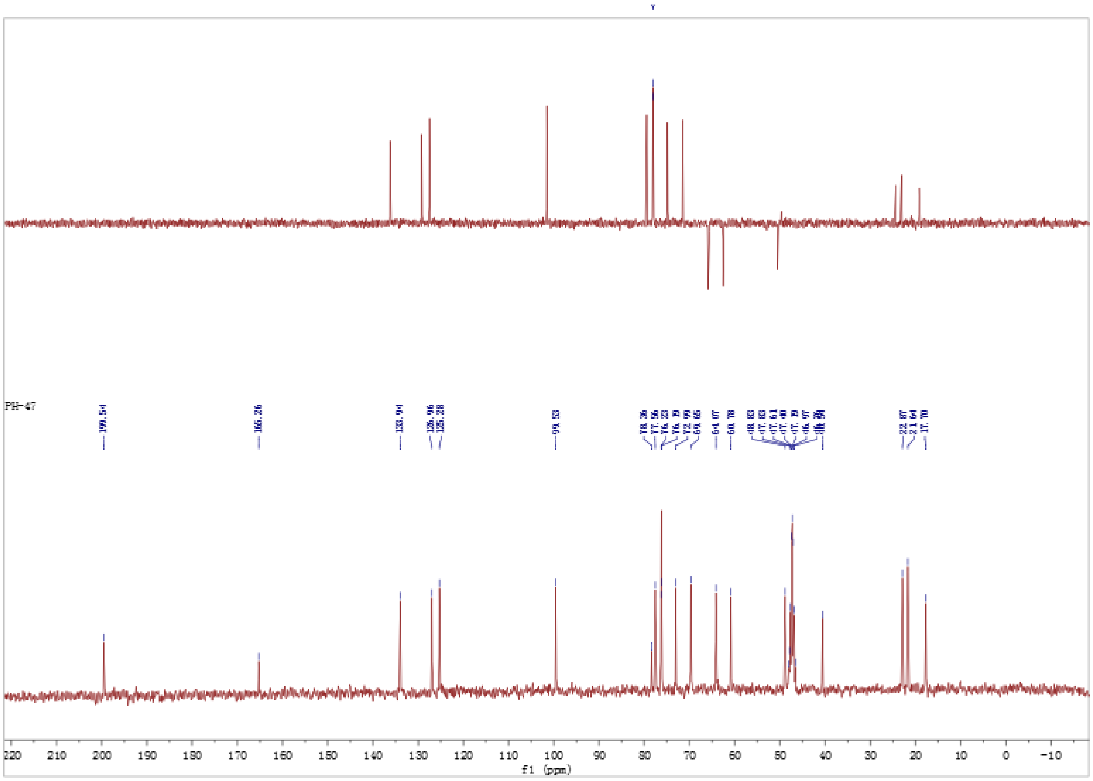

SI 28: ^{13}C-NMR and DEPT spectrum of compound **8**.

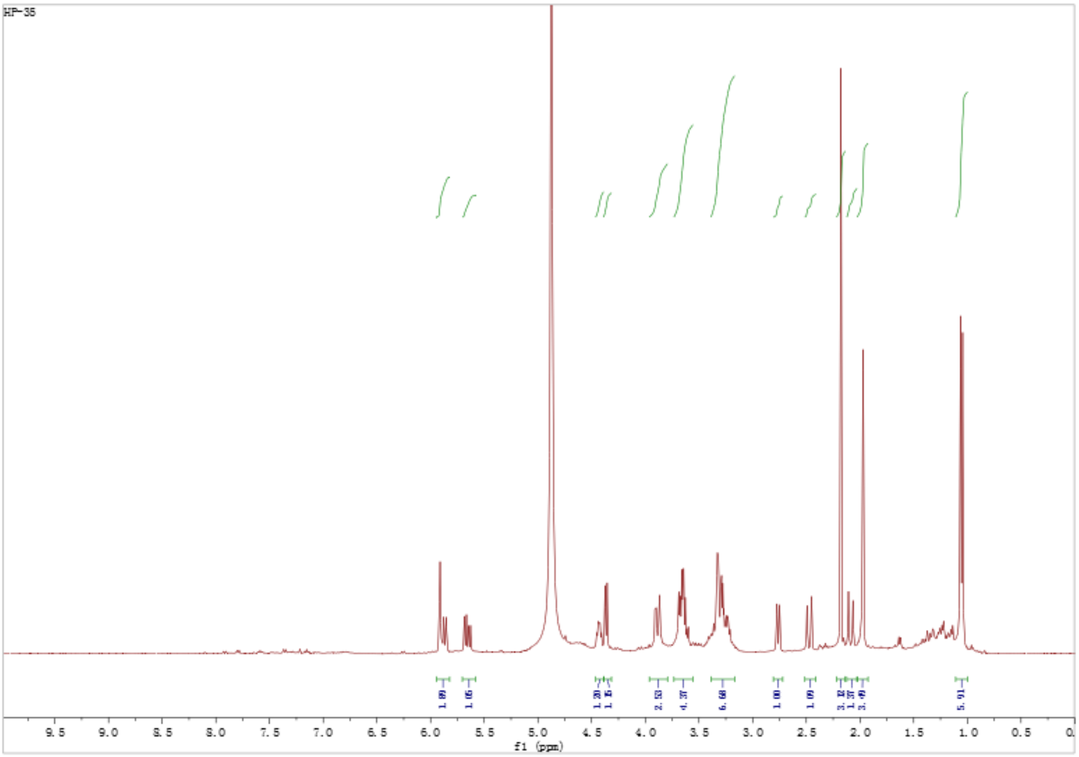

SI 29: ^{1}H-NMR spectrum of compound **9**.

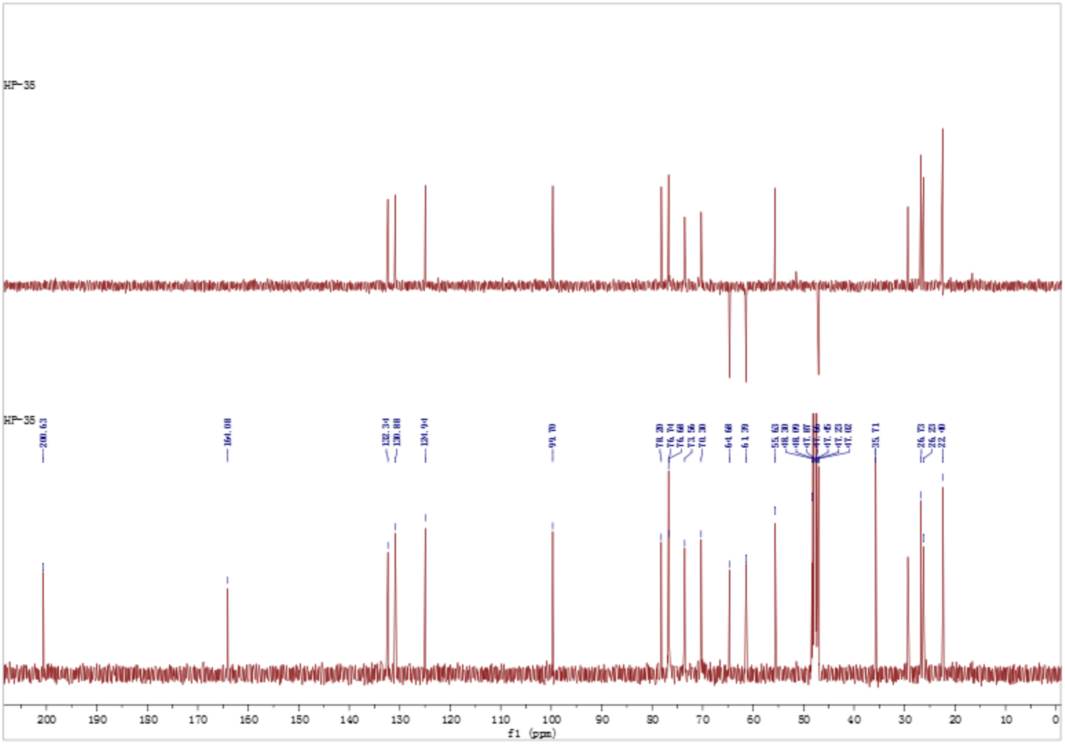

SI 30: ^{13}C-NMR and DEPT spectrum of compound **9**.

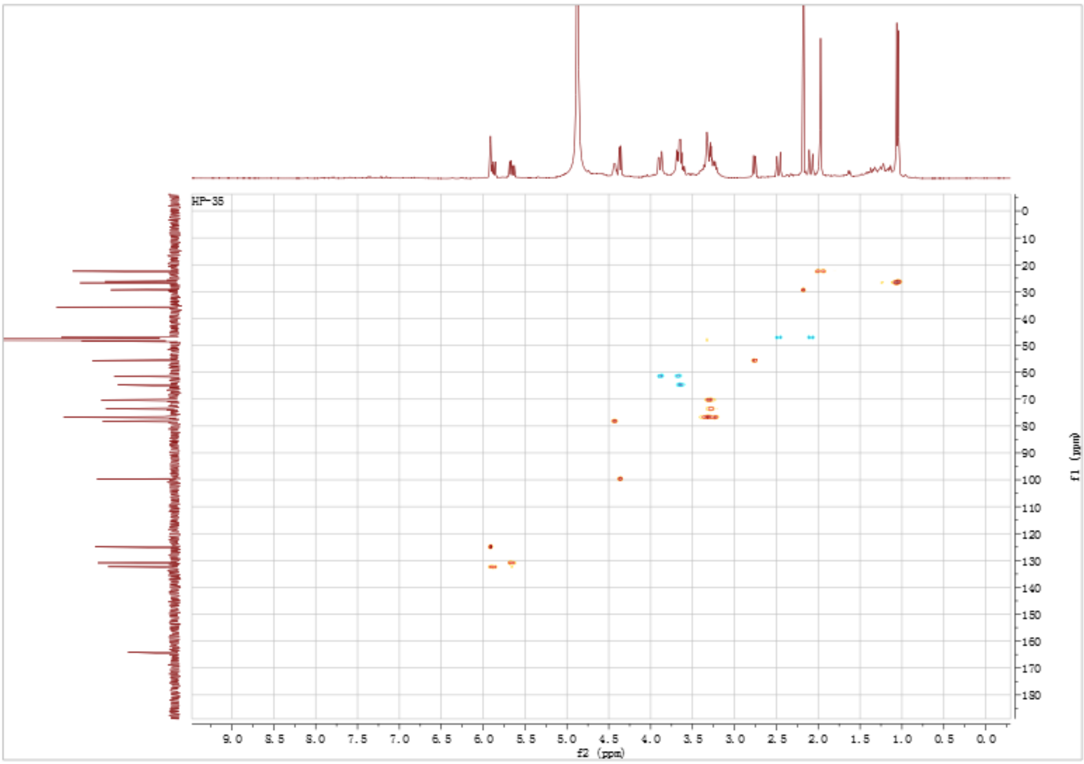

SI 31: HSQC spectrum of compound **9**.

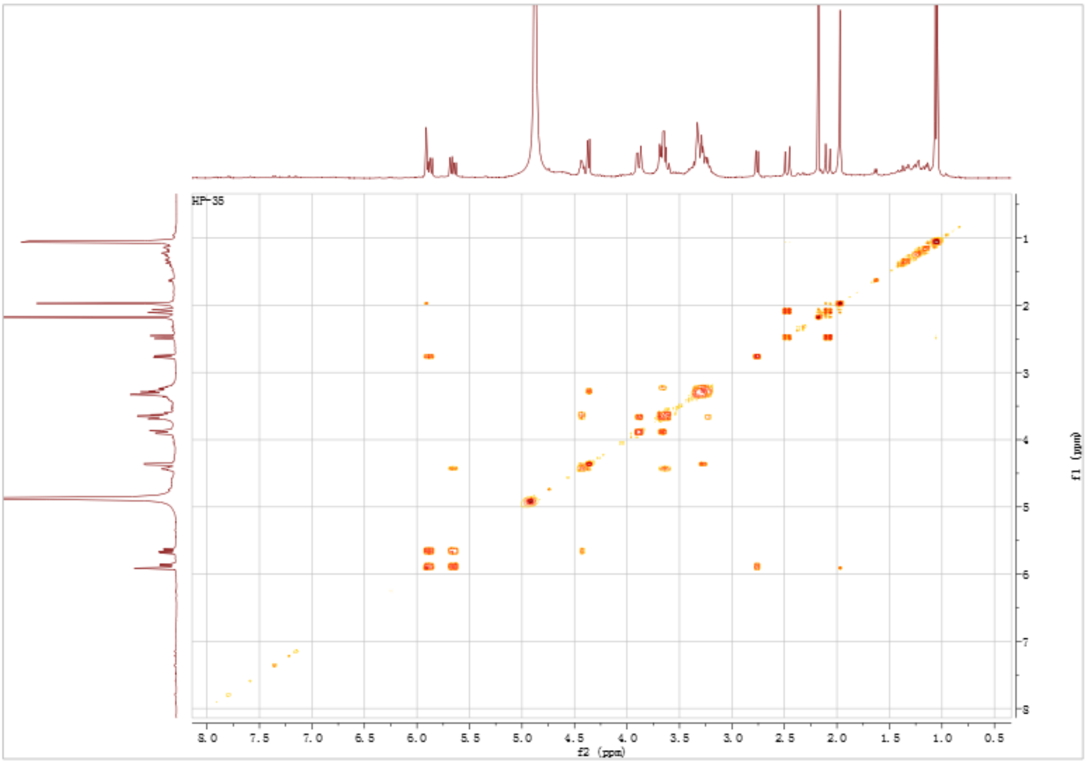

SI 32: ^{1}H-^{1}H COSY spectrum of compound **9**.

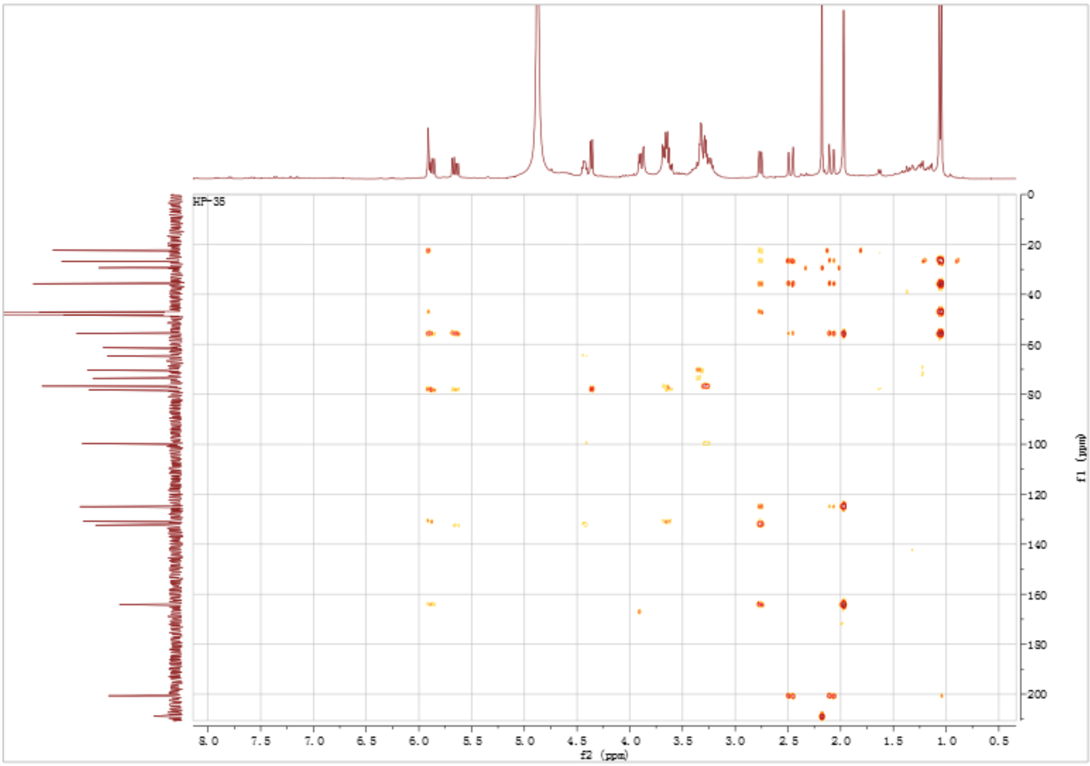

SI 33: HMBC spectrum of compound **9**

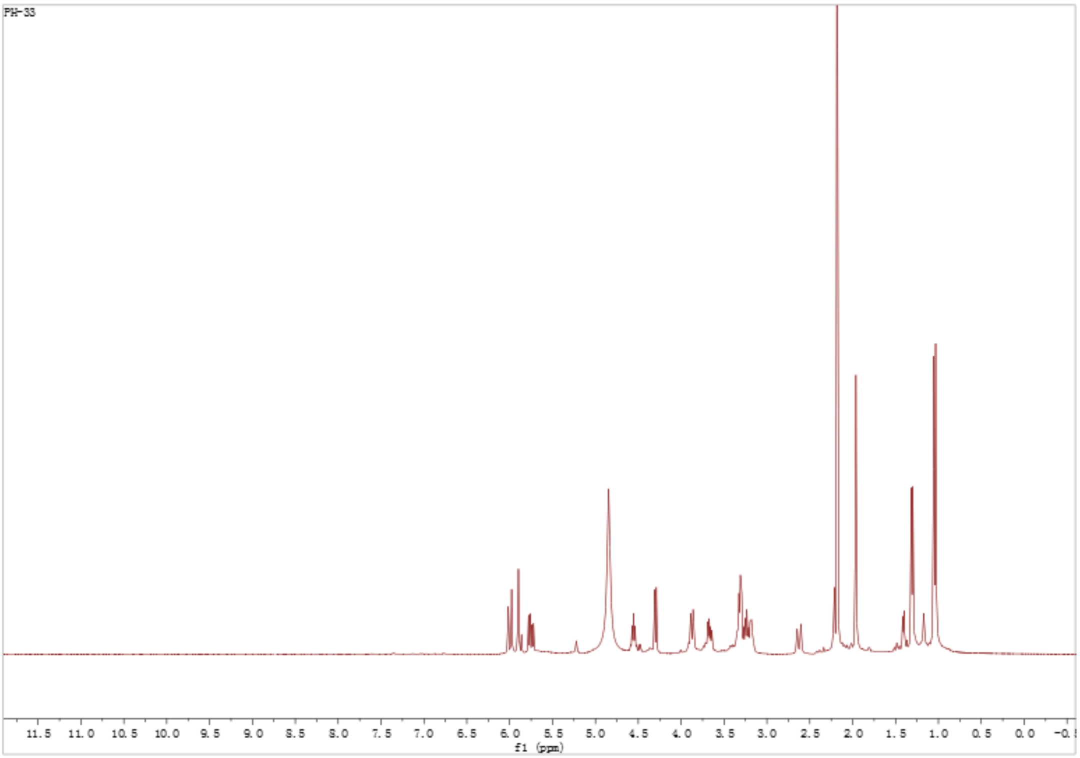

SI 34: ^{1}H-NMR spectrum of compound **10**.

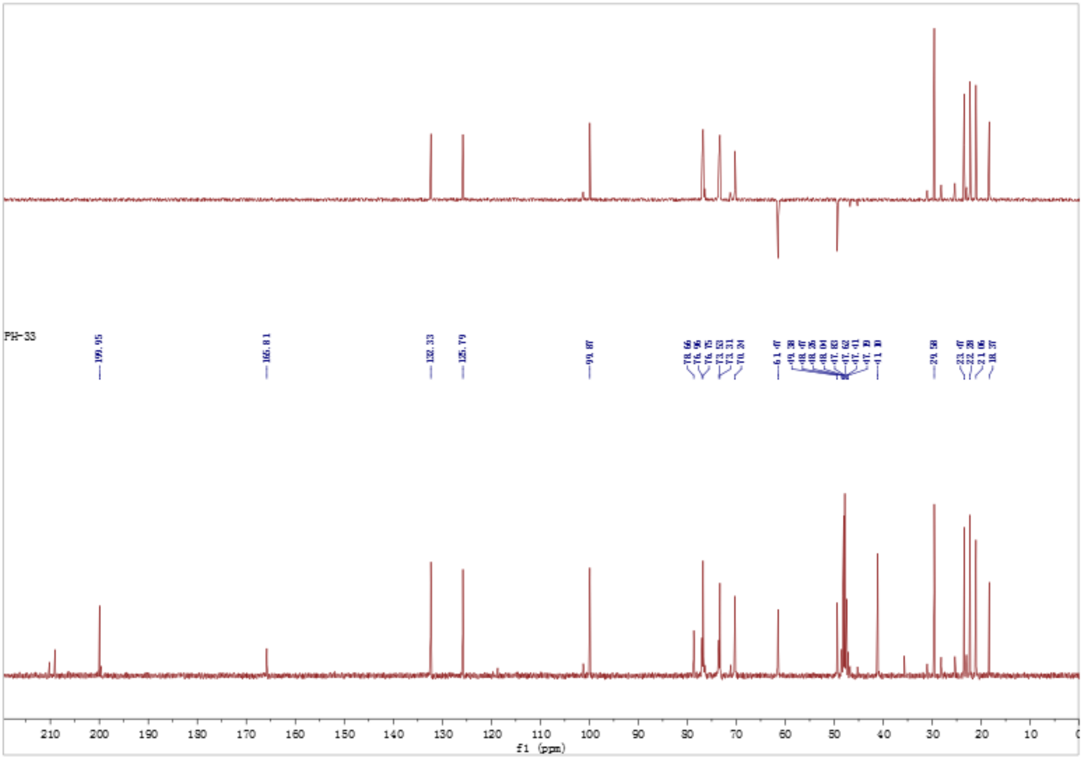

SI 35: ^{13}C-NMR and DEPT spectrum of compound **10**.

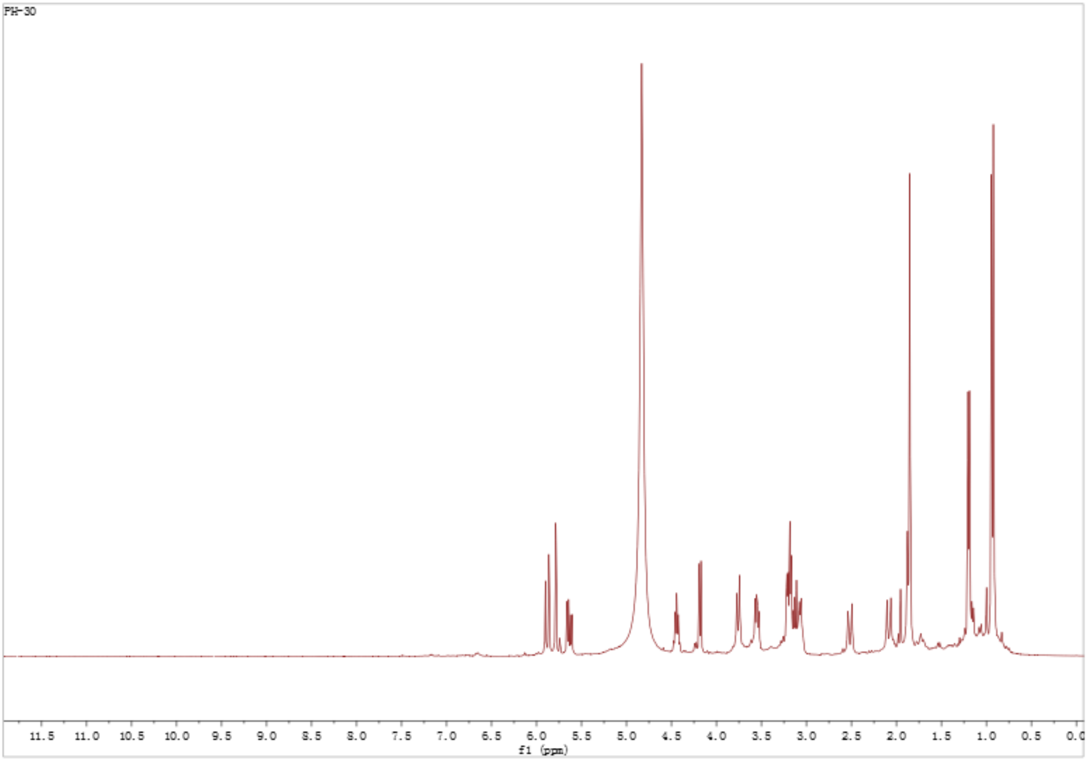

SI 36: ^{1}H-NMR spectrum of compound **11**.

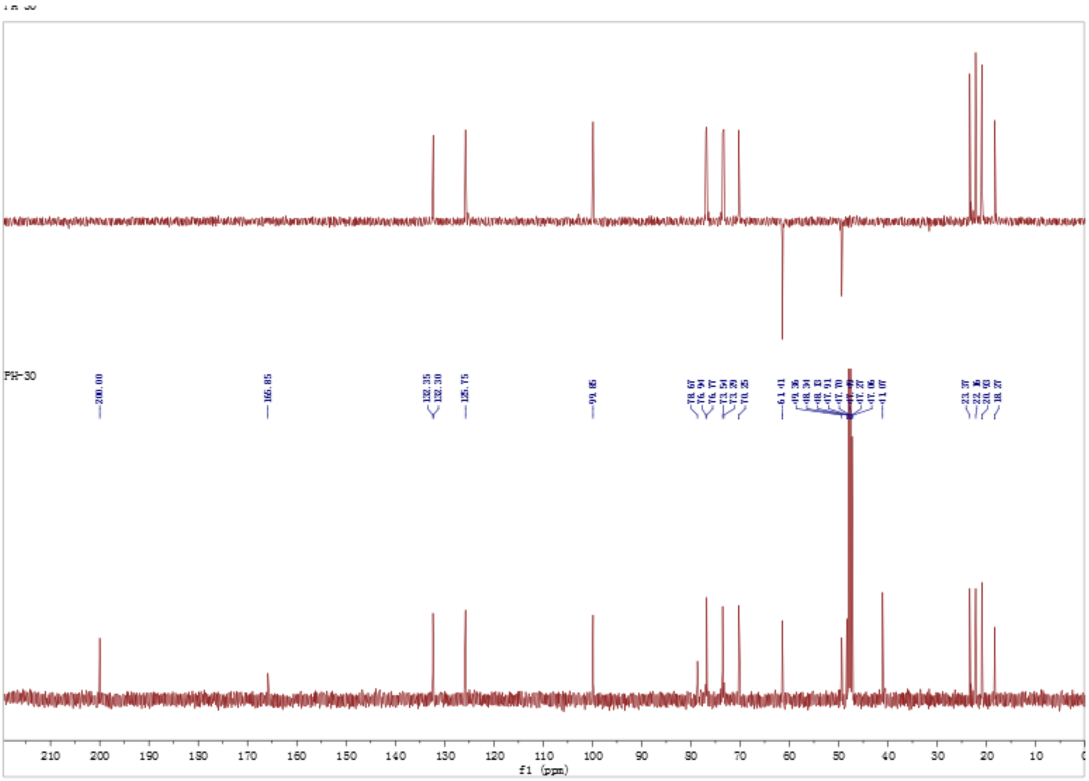

SI 37: ^{13}C-NMR and DEPT spectrum of compound **11**.

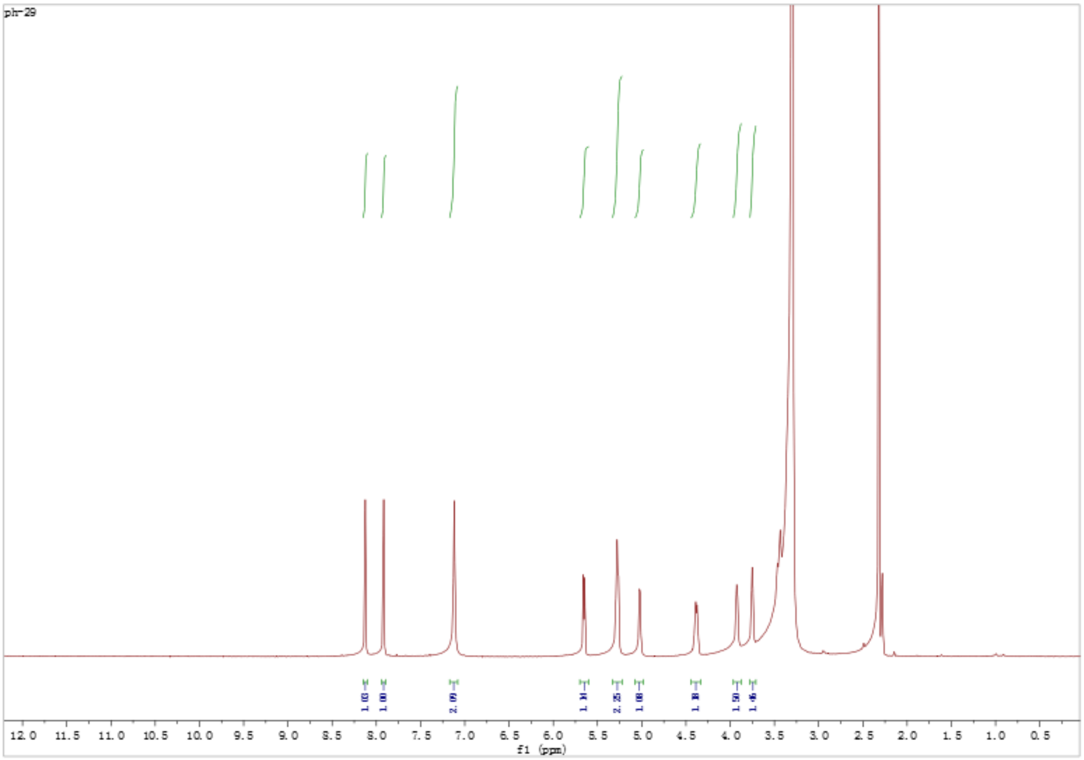

SI 38: ^{1}H-NMR spectrum of compound **12**.

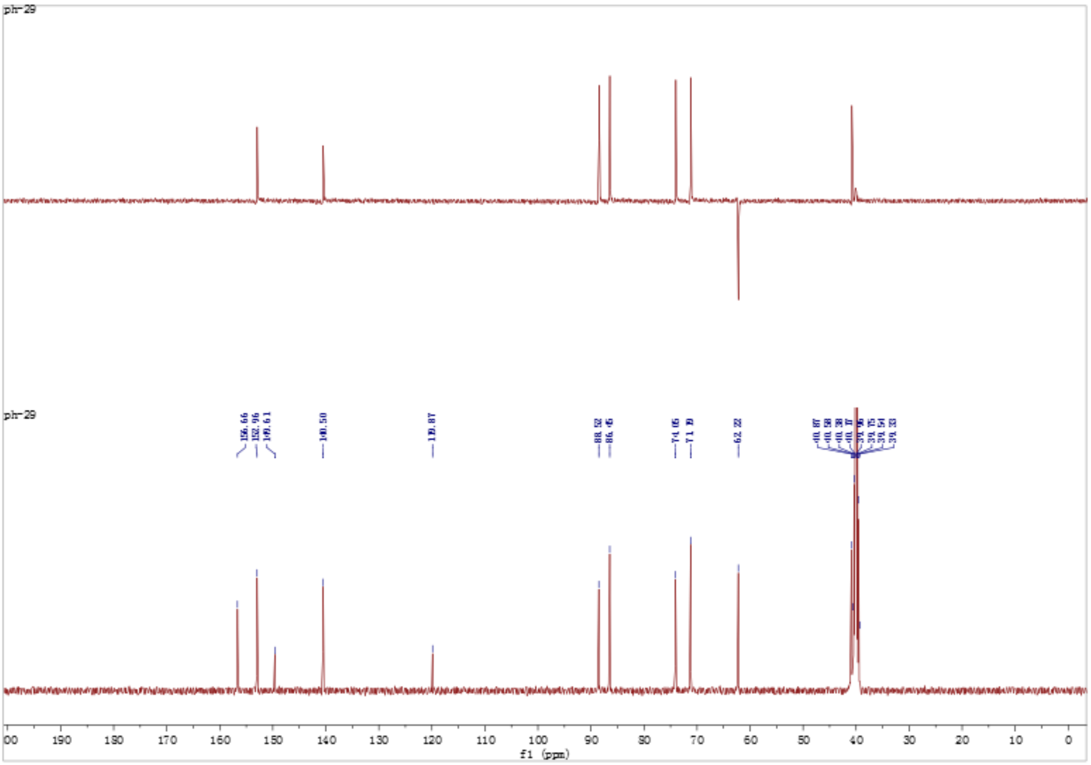

SI 39: ^{13}C-NMR and DEPT spectrum of compound **12**.

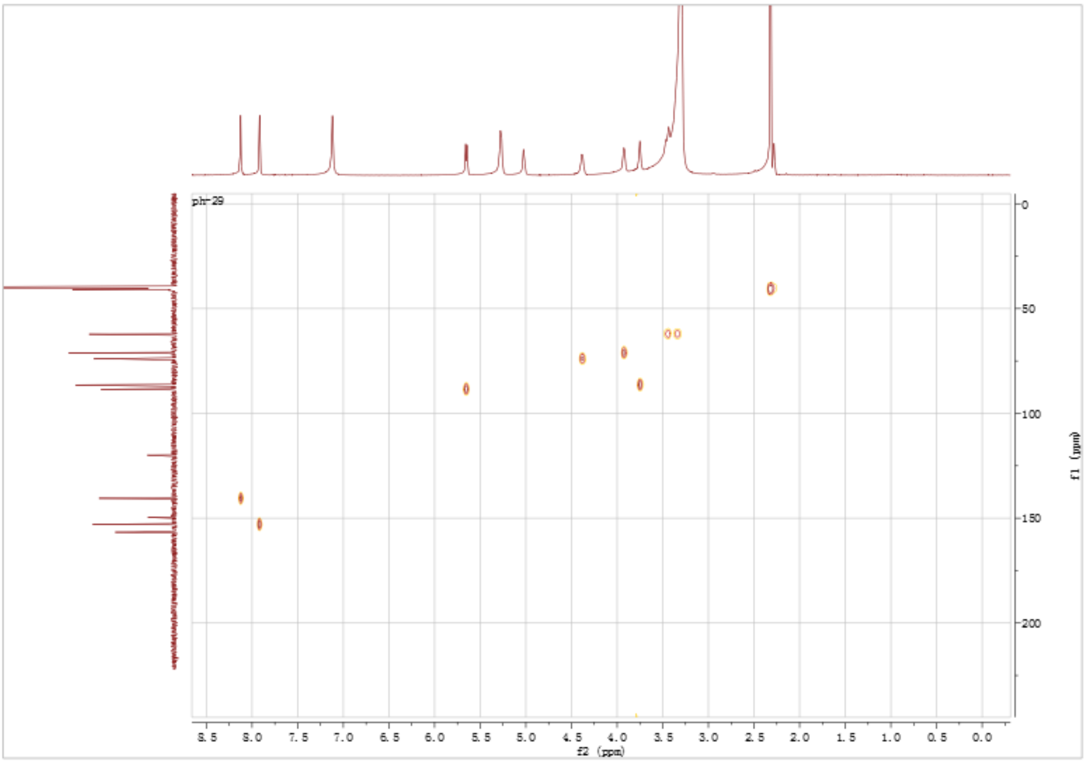

SI 40: HSQC spectrum of compound **12**.

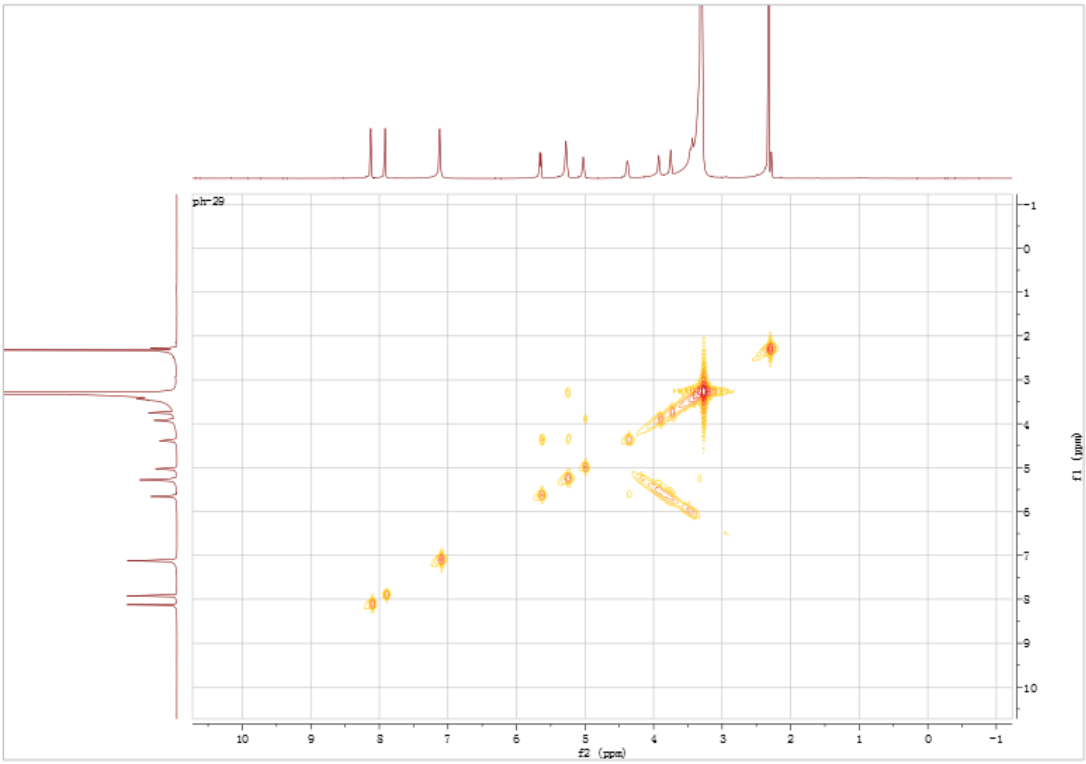

SI 41: ^{1}H-^{1}H COSY spectrum of compound **12**.

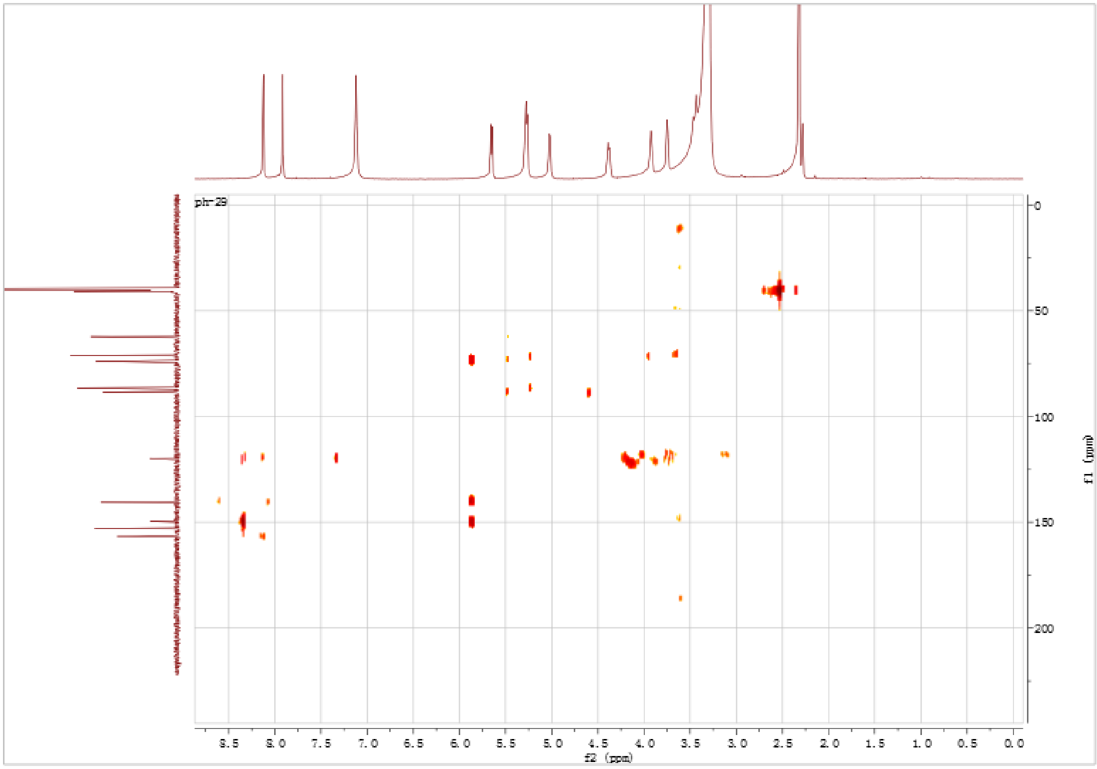

SI 42: HMBC spectrum of compound **12.**
